# Supplementary material for: Epigenome Mapping Reveals Distinct Modes of Gene Regulation and Widespread Enhancer Reprogramming by the Oncogenic Fusion Protein EWS-FLI1
Source: Cell Rep. 2015 Feb 19;10(7):1082–95. doi: 10.1016/j.celrep.2015.01.042 (PMC4542316; doi:10.1016/j.celrep.2015.01.042)
Supplement: Document S1. Supplemental Experimental Procedures, Figures S1–S5 [file mmc1.pdf]

Cell Reports

Supplemental Information

Epigenome Mapping Reveals Distinct Modes of Gene Regulation and Widespread Enhancer Reprogramming by the Oncogenic Fusion Protein EWS-FLI1

Eleni M. Tomazou, Nathan C. Sheffield, Christian Schmidl, Michael Schuster, Andreas Schönegger, Paul Datlinger, Stefan Kubicek, Christoph Bock, and Heinrich Kovar

SUPPLEMENTAL INFORMATION

Supplemental Table Legends

Table S1. Epigenome sequencing statistics, related to Figure 1 (provided as an Excel file)

Supplemental Figures

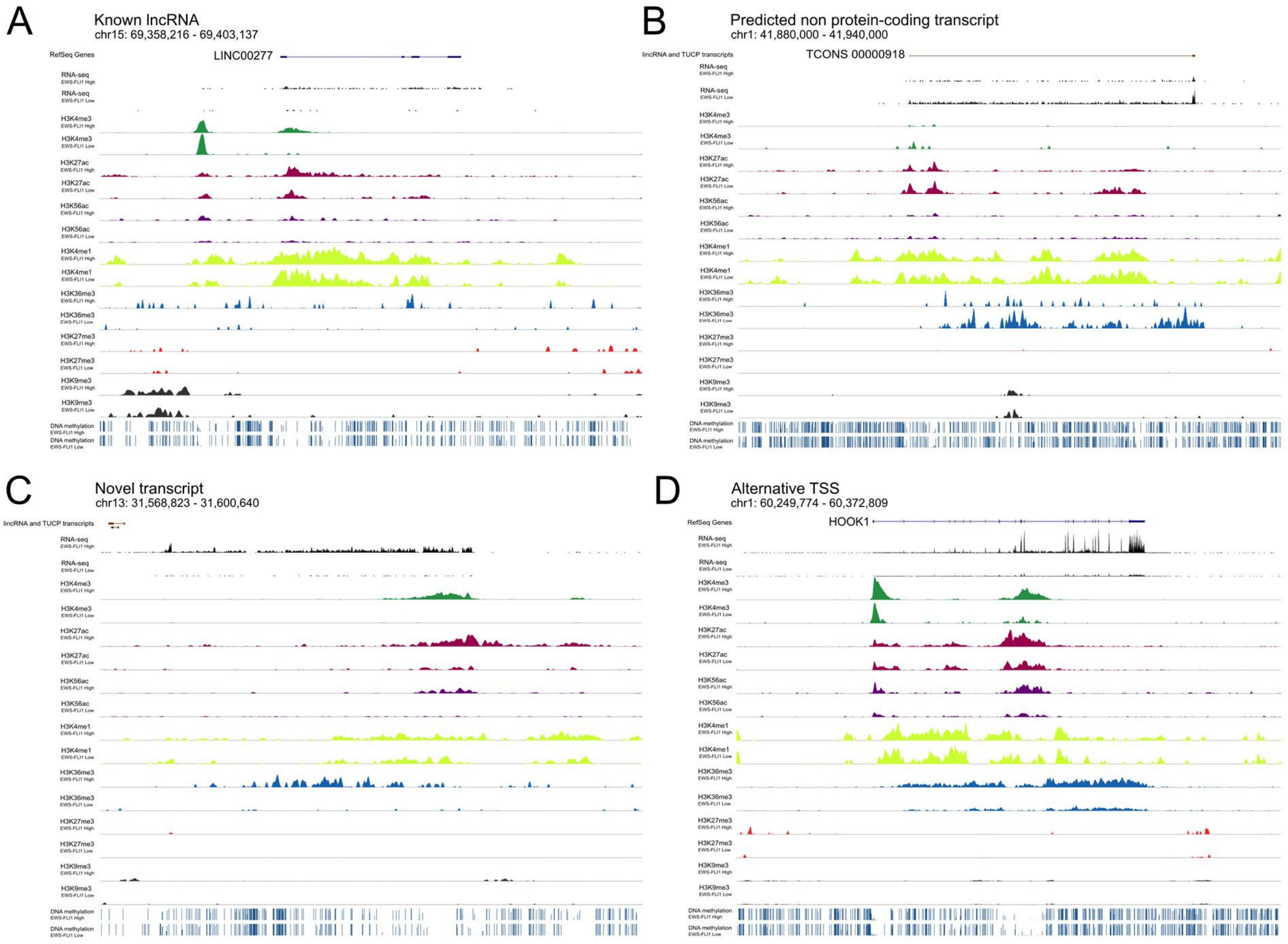

Figure S1: Epigenome maps for EWS-FLI1 regulated transcripts, Related to Figure 1

Genome browser screenshots of epigenome maps for the A673 cell line, showing the genomic loci of four EWS-FLI1 regulated transcripts: a known lincRNA (A); a predicted non-coding transcript (B); a novel transcript (C); and an alternative transcription start site of a known gene (D). All transcripts are differentially expressed between the EWS-FLI1 high state (before EWS-FLI1 knockdown) and the EWS-FLI1 low state (after EWS-FLI1 knockdown). This figure illustrates the use of chromatin maps for discovering EWS-FLI1 regulated transcripts. All data are available online at http://tomazou2015.computational-epigenetics.org.

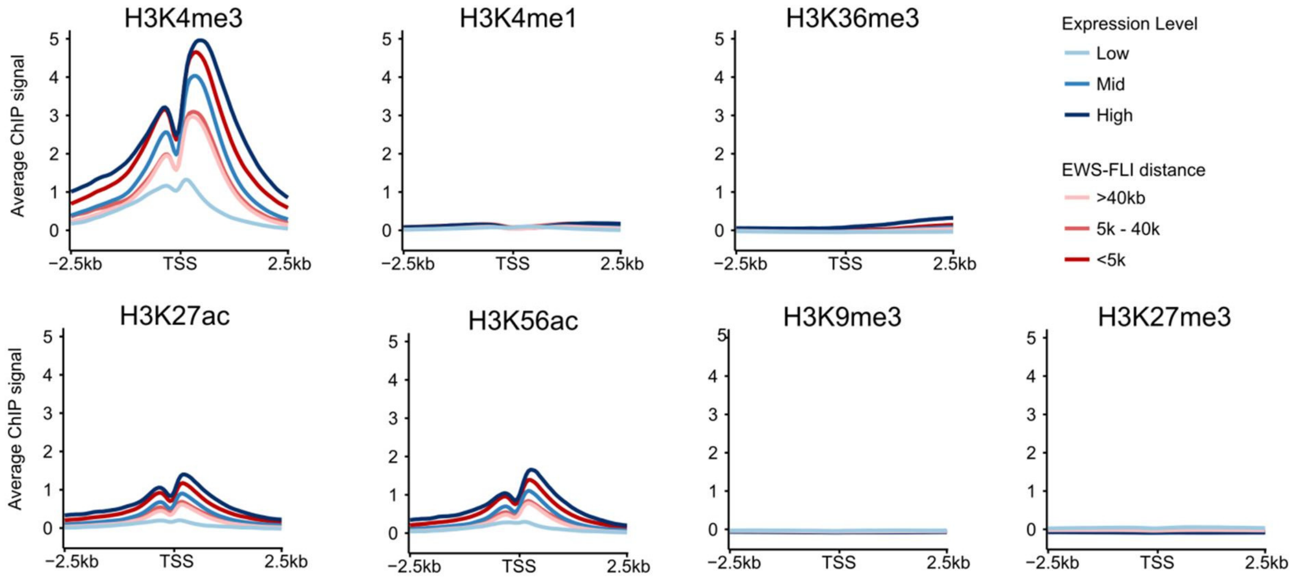

Figure S2: Distribution of histone modifications around transcription start sites, Related to Figure 2

Composite plots showing the aggregate ChIP-seq enrichment for each histone modification around the TSS, stratified in two ways: by the expression levels of associated genes (shades of blue); and by the distance to the nearest EWS-FLI1 binding peak (shades of red). These plots are the same as those shown in Figure 2A of the main manuscript, except that here the y-axis is scaled in the same way across all plots (which makes it easier to see the strong differences in observed ChIP-seq signal between different histone marks).

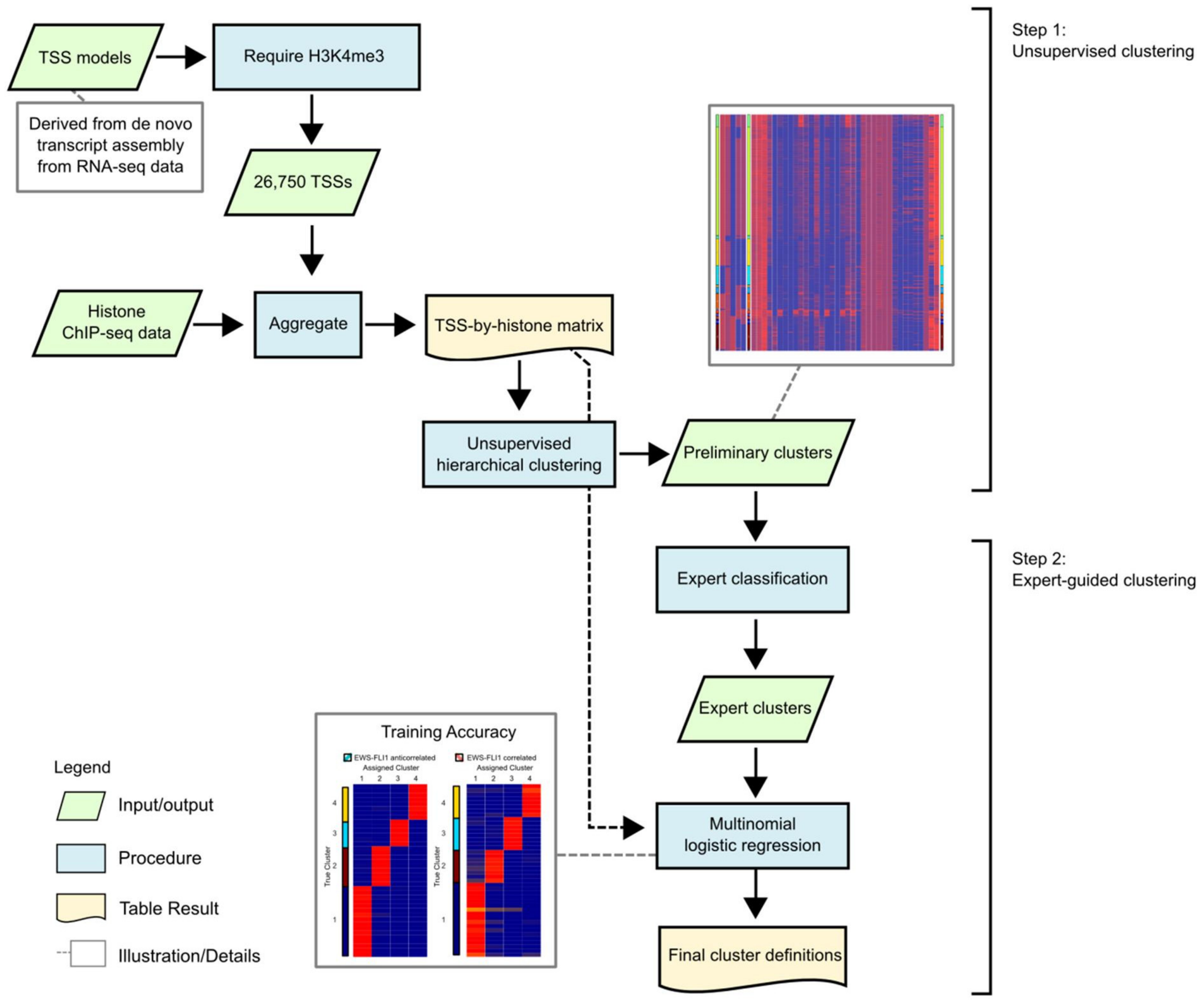

Figure S3: Workflow for chromatin-based clustering of EWS-FLI1 regulated transcripts, Related to Figure 3

Schematic diagram illustrating the semi-supervised clustering method that was used to group EWS-FLI1 regulated genes into clusters based on their histone marks. After annotating each TSS with the quantitative enrichment for promoter-associated histone marks, the TSSs were clustered by unsupervised hierarchical clustering. The resulting preliminary clusters were examined to define a set of expert examples, which provided the basis for training a multinomial logistic regression model. Using the model to predict cluster assignments on the original dataset resulted in the final cluster definitions. More details of the algorithm are given in the Supplemental Experimental Procedures.

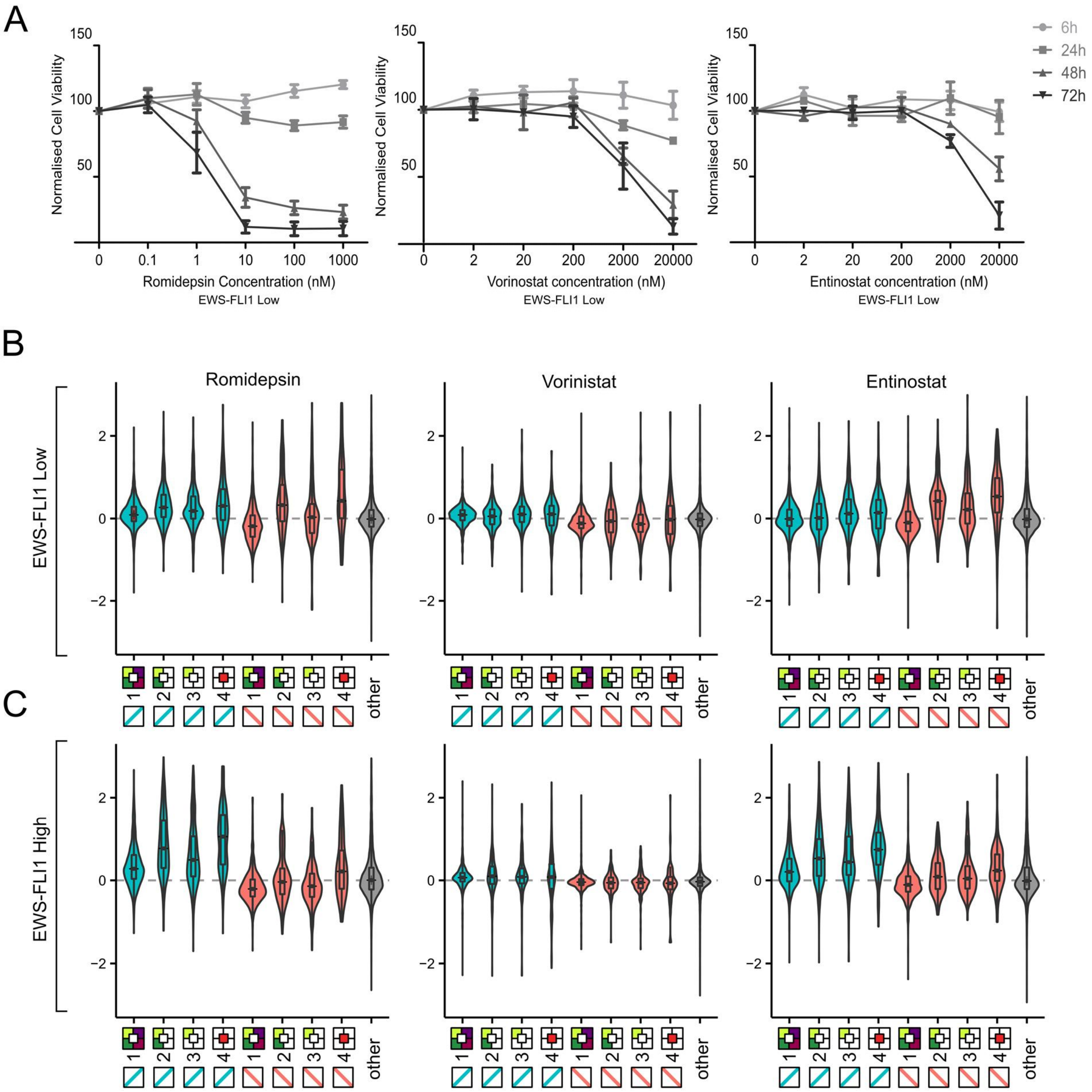

Figure S4: Response to treatment with HDAC inhibitors, Related to Figure 5

A: Dose-response curves showing the cell viability effect of three histone deacetylase inhibitors (HDACi) at different concentrations (x-axis) and time points (greyscale curves). Data are shown for the EWS-FLI1 low state. Cell viability was measured by the MTT assay and error bars represent the SEM of triplicate experiments.

B: Violin plots visualizing gene expression changes in response to HDAC inhibitor treatment in the EWS-FLI1 low state. The y-axis shows fold change, and the x-axis divides the genes by cluster and direction of response to EWS-FLI1 knockdown, as indicated by the annotation icons. Gray bars represent all other genes (those not responding to EWS-FLI1 knockdown).

C: Same as panel B, but for the EWS-FLI1 high state.

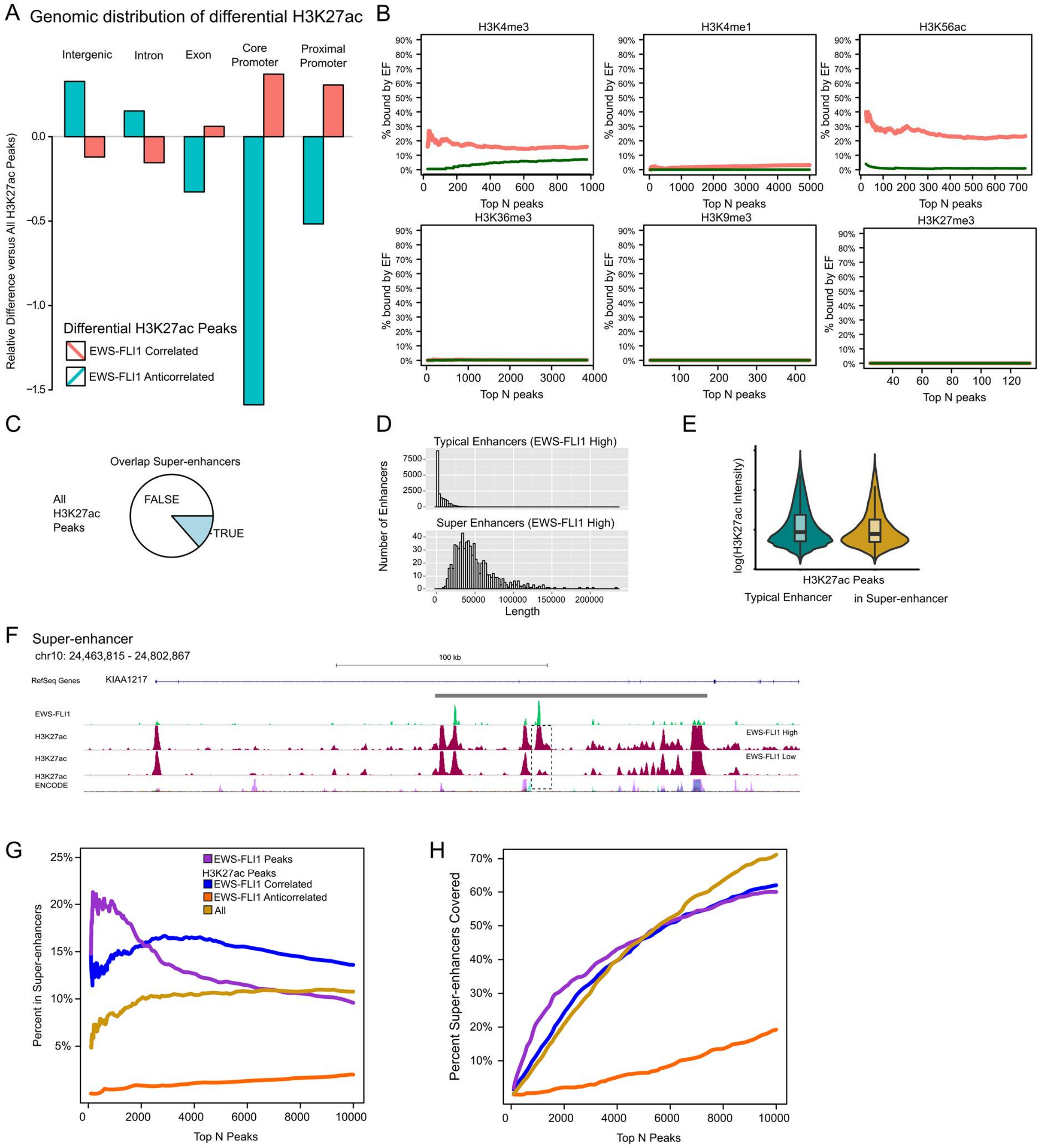

Figure S5: Enhancers & super-enhancers in EWS-dependent cells, Related to Figure 6

A: Bar charts showing the localization of differential H3K27ac peaks compared to non-differential H3K27ac peaks. The y-axis shows log fold changes indicative of relative enrichment or depletion.

B: Line charts analogous to Figure 6C, showing the histone marks other than H3K27ac. For each histone mark, differential peaks are called and then ranked by magnitude of change. These peaks are then tested for overlap with EWS-FLI1 binding site, and a curve showing the percentage of differential histone peaks covering an EWS-FLI1 binding site for the top N peaks is shown. Red line: EWS-FLI1 correlated; green line: EWS-FLI1 anticorrelated.

C: Pie chart illustrating the percentage of H3K27ac peaks that overlap with super-enhancers.

D: Histograms of enhancer and super-enhancer length distributions, showing that super-enhancers are longer than typical enhancers.

E: Violin plots showing that H3K27ac peaks in super-enhancers are in general not individually stronger than H3K27ac peaks located outside of super-enhancers.

F: Genome browser screenshot showing a super-enhancer (grey box) with a localized change in H3K27ac overlapping an EWS-FLI1 binding peak (dotted box). This peak is cell-type specific when compared with cross-tissue H3K27ac data from the ENCODE project (bottom track).

G: Line graph showing the percentage of peaks in super-enhancers for four sets of regions: All H3K27ac peaks (yellow), EWS-FLI1 correlated differential H3K27ac peaks (blue), EWS-FLI1 anticorrelated differential H3K27ac peaks (red), and EWS-FLI1 peaks (purple). Each set of regions is ranked by signal intensity (decreasing from left to right), and the y-axis shows the cumulative percentage of Top N peaks that overlap with a super-enhancer.

H: Line graph showing the percentage of super-enhancers that contain at least one of the top N peaks for each of the four sets of regions described for panel F.

Supplemental Website

The epigenome maps as well as the raw and processed data underlying the presented analyses are available online: http://tomazou2015.computational-epigenetics.org.

Supplemental Experimental Procedures

Cell Culture

We used a subclone of the Ewing sarcoma cell line A673 that has been stably transfected with a doxycycline-inducible shRNA against the EWS-FLI1 fusion protein (Carrillo et al., 2007). Cells were propagated in DMEM+GlutaMax supplemented with 10% fetal bovine serum, 50µg/ml Zeocin (InvivoGen, ant-zn-5p) and 2µg/ml Blasticidin (InvivoGen, ant-bl-10p). For the EWS-FLI1 shRNA induction, 1µg/ml doxycycline (Sigma, D9891) was added to the media for 53 hours. For the additional Ewing sarcoma cell lines TC252, SK-N-MC and STA-ET-7.2, the EWS-FLI1 knockdown was performed using transient transfection with shRNA constructs as previously described (Ban et al., 2008).

Bisulfite sequencing experiments

For reduced representation bisulfite sequencing (RRBS), 100 ng of genomic DNA were digested for 12 hours at 37°C with 20 units of MspI (New England Biolabs, R0106L) in 30 µl of 1x NEB buffer 2. Fill-in and A-tailing were performed by addition of Klenow Fragment 3’ > 5’ exo- (New England Biolabs, M0212L) and dNTP mix (10 mM dATP, 1 mM dCTP, 1 mM dGTP). After ligation to methylated Illumina TruSeq LT v2 adaptors using Quick Ligase (New England Biolabs, M2200L), the libraries were size selected by performing a 0.75x clean-up with AMPure XP beads (Beckman Coulter, A63881). The four libraries were pooled in equal amounts based on qPCR data and bisulfite converted using the EZ DNA Methylation Direct

Kit (Zymo Research, D5020) with changes to the manufacturer's protocol: conversion reagent was used at 0.9x concentration, incubation performed for 20 cycles of 1 min at 95°C, 10 min at 60°C and desulphonation time extended to 30 min. Bisulfite-converted libraries were enriched for 17 cycles using PfuTurbo Cx Hotstart DNA Polymerase (Agilent, 600412). After a 2x AMPure XP clean-up, quality control was performed by a Qubit dsDNA HS (Life Technologies, Q32854) and Experion DNA 1k assay (BioRad, 700-7107).

For whole genome bisulfite sequencing (WGBS), 3 µg of DNA were fragmented by Covaris S2 system two times for 60 seconds at duty cycle 20%, intensity 4, and 200 cycles per burst. Fill-in, A-tailing and adapter ligation were performed by using the TruSeq DNA sample preparation kit (Illumina) following the manufacturer's protocol. After each step fragments were purified by performing a 0.8x clean-up with AMPure XP beads (Beckman Coulter, A63881). Bisulfite conversion was performed by using the EpiTect Bisulfite kit (Qiagen) following the protocol specified for DNA isolated from FFPE tissue samples. Quantitative real-time PCR (20 µl reaction) was performed to determine the minimal PCR cycle number for library amplification using 1 µl bisulfite-converted DNA. Bisulfite-converted libraries were enriched for the determined number of cycles using PfuTurbo Cx Hotstart DNA Polymerase (Agilent, 600412). After a 2x AMPure XP clean-up, quality control was performed by a Qubit dsDNA HS (Life Technologies, Q32854) and Experion DNA 1k assay (BioRad, 700-7107).

ChIP-seq experiments

Chromatin immunoprecipitation (ChIP) was performed using the iDeal ChIP-seq kit (Diagenode) following the manufacturer's instructions. One million cells were cross-linked with 1% formaldehyde for 8 min at room temperature with constant agitation followed by quenching with 125 mM glycine for 5 min at room temperature. Nuclei were isolated and chromatin was sheared using the truChIP Low Cell Chromatin Shearing Kit (Covaris) and an S220 Ultrasonicator from Covaris. DNA fragments were in the range of 200-700 base pairs. Chromatin was incubated with antibody overnight at 4°C, with constant agitation. The following antibodies were used: H3K4me3 (Diagenode, C15410003-50, pAb-003-050), H3K27me3 (Diagenode, C15410195, pAb-195-050), H3K4me1 (Diagenode, pAb-194-050), H3K27ac (Diagenode, pAb-196-050), H3K56ac (Active Motif, 39281), H3K9me3 (Diagenode, pAb-193-050), H3K36me3 (Diagenode, pAb-192-050). During ChIP-seq library preparation, DNA isolated from ChIP experiments as well as input control DNA were end repaired, A-tailed, ligated to barcoded Illumina adaptors, PCR-amplified, and pooled for sequencing.

ATAC-seq

Open chromatin mapping was performed with the assay for transposase accessible chromatin (ATAC-seq) as previously described (Buenrostro et al., 2013) with minor adaptations for A673 cells. In each experiment, $1x10^5$ cells were washed once in 50 µl PBS, resuspended in 50 µl ATAC-seq lysis buffer (10 mM Tris-HCl, pH 7.4, 10 mM NaCl, 3 mM $MgCl_2$ and 0.1% IGEPAL CA-630), and centrifuged for 10 min at 4 °C. Upon centrifugation, the pellet was washed briefly in 50 µl $MgCl_2$ buffer (10mM Tris pH8.0, 5mM $MgCl_2$) before incubating in the transposase reaction mix (25 µL 2× TD buffer, 2.5 µL transposase (Illumina) and 22.5 µL nuclease- free water) for 30 min at 37°C. After DNA purification with the MinElute kit (Qiagen), 1 µl of the eluted DNA was used in a qPCR reaction to estimate the optimum number of amplification cycles. Library amplification was followed by a SPRI size-selection to exclude

fragments larger than 1200bp. DNA concentration was measured with a Qubit fluorometer (Life Technologies).

RNA-seq experiments

Total RNA was isolated with TRIzol Reagent (Invitrogen). RNA amount was measured using Qubit 2.0 Fluorometric Quantitation system (Life Technologies), and RNA integrity number (RIN) was determined using Experion Automated Electrophoresis System (Bio-Rad). RNA-seq libraries for the reference transcriptomes of EWS-FLI1 high and EWS-FLI1 low cells were prepared using TruSeq Stranded Total RNA LT Ribo-Zero Human/Mouse/Rat sample preparation kit (Illumina). RNA-seq libraries for samples treated with HDAC inhibitors were prepared using a Sciclone NGS Workstation (PerkinElmer) and a Zepyhr NGS Workstation (PerkinElmer) with the TruSeq Stranded mRNA LT sample preparation kit (Illumina). Library amount and quality was determined using Qubit 2.0 Fluorometric Quantitation system (Life Technologies) and Experion Automated Electrophoresis System (Bio-Rad).

Western blotting

Western blots were carried out using standard protocols and with the same antibodies that were also used for the ChIP-seq experiments. In addition, antibodies for EWS-FLI1 (MyBio-Source, MBS300723) and beta Actin (Abcam, 8226) were used. Western blot signals were acquired with an Odyssey Li-COR laser scanning and imaging system (Li-COR).

Drug treatment experiments

Cell viability was measured by performing MTT colometric assay. A673 cells were resus-pended in fresh media containing one of the below drugs: Romidepsin (0.1 nM, 1 nM, 10 nM, 100 nM, and 1,000 nM), vorinostat (2 nM, 20 nM, 200 nM, 2,000 nM), entinostat (2 nM, 20 nM, 200 nM, 2,000 nM, and 20,000 nM) or vehicle (DMSO, 0.1%). Cells were plated in 96-well plates at 10,000 cells/well in a volume of 100 µl. Viability was measured after 6, 24, 48, and 72 hr incubations by addition of MTT solution (20 µl, 5 mg/ml) (Sigma, M2128) and lumi-nescence measurement on an EnSpire multimode plate reader. For the EWS-FLI1 low state, inhibitors were added 24h after adding doxycycline to induce knockdown. All experiments were performed in three biological replicates (each biological replicate is the average of four technical replicates). Based on the dose-response curves, the following drug concentrations and time points were selected for measuring the transcriptome response by RNA-seq in two biological replicates: Romidepsin (1nM) for 24h, vorinostat (200 nM) for 24h, entinostat (2,000 nM) for 6h or vehicle (DMSO, 0.1%). Drugs were provided by the Platform Austria for Chemical Biology (PLACEBO) at the CeMM Research Center for Molecular Medicine of the Austrian Academy of Sciences.

Sequencing and initial data processing

Sequencing was performed on the Illumina HiSeq 2000 platform. Base calls provided by the Illumina Realtime Analysis software were converted into BAM files using Illumina2bam (https://github.com/wtsi-npg/illumina2bam) and subsequently demultiplexed using BamIn-dexDecoder from the same package. Initial quality control was performed using the FastQC

software (http://www.bioinformatics.babraham.ac.uk/projects/fastqc/). All bioinformatic analyses were relative to the hg19/GRCh37 assembly of the human genome.

DNA methylation data processing

WGBS reads were aligned using Bismark (Krueger and Andrews, 2011), whereas RRBS reads were aligned with BSMAP/RRBSMAP (Xi et al., 2012). Preprocessing of the sequenced reads comprising quality trimming and adapter removal was done with Trimmomatic (Bolger et al., 2014) in both cases, and DNA methylation calling was done with Bismark for WGBS data and with Bis-SNP for RRBS data (Liu et al., 2012). We measured the concordance between biological replicates by dividing the genome into 1-kilobase tiling regions and calculating the correlation across replicates of the average DNA methylation level in each region. We then combined WGBS and RRBS to obtain a set with high coverage in CpG islands as well as uniform baseline coverage genome-wide. With this combined dataset for both the EWS-FLI1 high and the EWS-FLI1 low state, we calculated total CpG coverage for different region types: (i) a set of CpG Islands obtained from the UCSC Genome Browser; (ii) promoter regions defined as the 2 kilobases upstream of TSSs according to our gene model analysis (Figure S3); and (iii) 1-kilobase tiles throughout the genome. To estimate average coverage, for each region set we summed the number of reads covering each CpG and then divided by the number of CpGs in the region. RnBeads (Assenov et al., 2014) was used for quality control and initial analysis of the DNA methylation data (Bock, 2012), while all additional analyses were performed in R (http://r-project.org/)

Histone modification data processing

ChIP-seq reads were aligned with Bowtie2 (Langmead et al., 2009). MACS2 (Zhang et al., 2008) was used for peak calling in each biological replicate with a pooled input dataset as baseline. The MACS2 bdgcmp tool was used in 'subtract' mode to plot a signal track for visual inspection via a UCSC Genome Browser track hub. Finally, the R/Bioconductor package diffBind (http://www.bioconductor.org/packages/release/bioc/html/DiffBind.html) was used in combination with custom R scripts to identify peak regions that show significant differences in binding occupancy. To assess the relationships among the histone marks, we extracted average signal scores in 1-kilobase tiling regions across the genome, and hierarchically clustered all the histone data.

ATAC-seq data processing

ATAC-seq reads were aligned with Bowtie2 (Langmead et al., 2009). After shifting the reads as described in the original protocol (Buenrostro et al., 2013), we created smoothed density tracks and called peaks using dipPeak (http://dippeak.computational-epigenetics.org), a kernel density estimator.

Gene expression data processing

RNA-seq reads were aligned with TopHat2 (Kim et al., 2013). We used the Ensembl transcript set (Homo sapiens, e73, September 2013) as reference transcriptome and processed each biological replicate separately. Cufflinks (Trapnell et al., 2013) was used to assemble transcripts from the spliced read alignments, again with the Ensembl transcriptome as refer-

ence. We allowed *de novo* assembly of transcript models in a first round of the analysis. The resulting custom transcript and gene set served as the basis throughout the rest of the study. When assessing differential expression with Cuffdiff, the RNA-seq of all relevant biological replicates were combining using Cuffmerge. Finally, the R/Bioconductor packages cummerbund (http://www.bioconductor.org/packages/release/bioc/html/cummeRbund.html) and biomaRt (http://www.bioconductor.org/packages/release/bioc/html/biomaRt.html) were used in custom R scripts to perform quality assessment and to refine the analysis results. In a second stage of the analysis, we profiled the transcriptomes after drug treatment. To keep the set of transcripts consistent throughout the analysis, the assembled and merged transcripts of the first stage were used as the reference for the Cufflinks transcriptome assembly step, this time suppressing de novo transcript assembly.

Composite histone signal

For Figure 2 and Figure S2, we aggregated histone ChIP-seq signal across regions centered at TSSs and at EWS-FLI1 binding sites. We extracted ChIP-seq signals for each histone mark in 5-kilobase windows surrounding each TSS. We divided TSSs in two ways: first, by expression level (high: top 20%; low: bottom 10%; mid: everything else); and second, by distance to the nearest EWS-FLI1 binding site (proximal: <5kb; distal: 5kb-40kb; none: >40kb). Cutoffs of 5kb and 40kb are reasonable arbitrary values chosen to roughly balance the number of genes in each category. We expect these cutoffs to capture the difference between binding sites that are obviously tied to a particular TSS (<5kb away) versus those that are still reasonably close, but less clear in their TSS assignment and more likely to be associated with distal histone modifications. We then plotted aggregate signal intensity surrounding the TSS for each of these groups of TSSs. To assess histone patterns at EWS-FLI1 binding sites, we aggregated ChIP-seq signal centered on the binding sites instead of the TSSs, dividing the sites by the distance to the nearest TSS.

Clustering genes based on epigenetic profile

To consider genes from the perspective of transcription start sites, we started with the TSS models output from cuffmerge (as described above). Because we have additional data about transcription initiation through the histone marks (H3K4me3), which is not considered by cufflinks, we first filtered the cufflinks TSSs by requiring that there be a MACS2-called peak of H3K4me3 in either the EWS-FLI1 high state or the EWS-FLI1 low state within 2.5 kilobases of the TSS. This produced a high-confidence set of 26,750 TSSs that are active in at least one of the two conditions we considered.

We then annotated each TSS for the status of each histone mark by assigning a single score summarizing the strength of each histone mark at each TSS. For histone marks generally found in core promoters (H3K4me3, H3K27ac, H3K56ac), we took the maximum peak score within a 1-kilobase window symmetrically surrounding the TSS. For H3Kme1, which is known to be more dispersed surrounding the TSS (Calo and Wysocka, 2013), we used a larger window of 5 kilobases. We also assessed H3K27me3 in 1kb windows at TSSs for this matrix. This resulted in a matrix of genes-by-modifications, and these five histone marks were used to cluster the TSSs.

We used a two-step approach to define the clusters. In the first step, we used an unsupervised hierarchical clustering on a weighted discrete version of the histone scores. We first

discretized the scores into 3 classes: absent (no peak present; class 0), peak (class 1), or strong peak (at least 2 standard deviations above average; class 2). H3K27me3 was restricted to 2 classes, as the intensity of this signal is assumed to be less relevant than its presence/absence. We assigned the absent class a score of 0, peaks to 1.5, and strong peaks to 2, and then weighted these discrete scores as follows: H3K27me3: 3; H3K4me3: 1.5; H3K4me1: 1; H3K56ac: 1; H3K27ac: 1. We observed that this weighting strategy assigns biological relevance to the histone marks, resulting in biologically meaningful clusters; for example, the heavy weight on H3K27me3 encourages the algorithm to preferentially cluster TSSs with this mark together.

To assess the strength of these cluster assignments, we fed the result of the unsupervised hierarchical clustering into a multinomial logistic regression. We used the glmnet R package, using a leave-one-out cross-validation to select a lambda. This effectively assigned a confidence score to each cluster assignment. We then selected a few of the top-scoring TSSs from each cluster to examine, and then manually classified these genes into "expert clusters". During the course of manual classification, we settled on four distinct clusters corresponding to four regulation modes. We classified 40-50 genes from each of the two experimental conditions, resulting in about 8-12 example genes for each expert-assigned cluster (Supplemental Website).

We used these expert-assigned cases to build models of the four regulation clusters we had observed. We used the same multinomial logistic regression strategy described above, fitting a lasso model to the expert examples. The training set performs almost perfectly in the cross-validation steps used to fit the model, implying high confidence in the cohesion of the clusters (Figure S3). We then used this model to predict the cluster assignment of the remaining genes that were not evaluated by the expert cluster assignment. We set a differential expression q-value cutoff of 0.2, and predicted cluster assignment for all genes that met this threshold. The confidence of the resulting predictions is general, and to further refine the list, we restrict the final set to only genes that are predicted to belong to a single class with at least 95% probability. This procedure was repeated independently for EWS-FLI1 correlated and EWS-FLI1 anticorrelated genes; with the correlated genes being clustered on the basis of their histone patterns in the EWS-FLI1 low state, and the anticorrelated genes on the basis of their histone patterns in the EWS-FLI1 high state.

Annotating clusters for enrichment

To identify any functional similarities within each cluster of genes we identified, we developed a comprehensive enrichment analysis tool that detects significant overlaps between a given set of genes (and their promoter regions) and several manually curated databases, including MSigDB (Liberzon et al., 2011), Cistrome (Liu et al., 2011), DNase hypersensitivity (Sheffield and Furey, 2012; Sheffield et al., 2013), and ENCODE transcription factor binding datasets (ENCODE Project Consortium, 2012). For this analysis we employed two different types of comparison: First, using a more traditional enrichment analysis, we compared sets of gene names to gene name sets in MSigDB. We also developed a location-based enrichment tool, Location Overlap Analysis (LOLA; http://lola.computational-epigenetics.org), to allow us to compare loci of interest to published sets of genomic regions, such as ChIP-seq results. In this setting, we avoided assigning TSSs to specific gene names, instead considering just the location of the TSS and looking for enrichment of other datasets within these regions. For the location-based enrichments, we tested for significant overlap with several hand-curated region set databases. We downloaded data from Cistrome, ENCODE transcription factor ex-

periments, and ENCODE DNase hypersensitivity tissue-specificity assignments. We used Fisher's exact tests to obtain p-values for each comparison, and ranked comparisons by p-value to identify the gene sets with most significant overlap.

Using this enrichment tool, we searched for significant enrichments in three different comparisons. First, we compared our cluster-assigned genes to the entire set of genes identified by the RNA-seq analysis. This resulted in general Ewing sarcoma genes, confirming that our differential genes match previous definitions of changes induced by EWS-FLI1 (Supplemental Website). Second, we searched for enrichments in each cluster versus genes in the other clusters; in other words, we restricted the universe of possible genes to only those regulated by EWS-FLI1. This identified cluster-specific effects (Figure 4; Supplemental Website). Finally, we tested for enrichments for the EWS-FLI1 correlated genes against EWS-FLI1 anticorrelated genes by restricting the universe of possible genes to those regulated in either direction within the same cluster (Figure 4; Supplemental Website).

Enhancers, super-enhancers, and tissue-specificity

To assess the genomic context of differential H3K27ac peaks, we used the set of differential H3K27ac peaks created by diffBind. We defined promoters as 2 kilobases upstream of TSSs, with TSSs defined by our merged annotation based on the RNA-seq data we generated. We then annotated the peaks as promoters if they overlapped an H3K4me3 peak; otherwise, we classified them as distal enhancer elements, because H3K27ac is commonly used as the defining mark for active enhancers.

We used ROSE to search for super-enhancers based on our H3K27ac data (Loven et al., 2013; Whyte et al., 2013). We assembled a set of super-enhancers for each of the two replicates of EWS-FLI1 expressing cells (EWS-FLI1 high state). To assemble a final list of super-enhancers we combined the replicates by requiring that a super-enhancer is present (overlapping) in both replicates. We used these definitions of super-enhancers to test for overlap with differential H3K27ac peaks. After defining super-enhancers, we subdivided the set of enhancers into those that overlap a super-enhancer, and those that overlap an EWS-FLI1 binding peak. After defining super-enhancers, we annotated them for nearby genes. We listed all genes within 50 kilobases of the super-enhancer, and selected a few promising candidates to highlight in the main figure. To assess tissue specificity, we used DNase hypersensitivity data from 72 human cell types published previously (Sheffield et al., 2013). For each differential H3K27ac peak, we calculated the number of samples that have a normalized DNase hypersensitivity score above a cutoff (0.4) within the H3K27ac peak. We then averaged this value across all peaks of a certain type, and looked at the relationship between average values. To calculate p-values, we used Kolmogorov-Smirnov tests on the distribution of average tissue-specificities.

DNA methylation and EWS-FLI1 binding

We divided H3K27ac peaks into those bound or not bound by EWS-FLI1. We then compared the distribution of average DNA methylation level between these sets of regions. We repeated this test for both EWS-FLI1 high and EWS-FLI1 low conditions, and found similar results. To confirm, we next compared DNA methylation signal before and after EWS-FLI1 knockdown. We considered all H3K27ac peaks with at least 25 total measurements of CpG methylation in each condition. We used a Fisher's exact test to identify significant difference be-

tween the conditions, and then looked at the absolute difference in methylation between the conditions for those with significant difference. For the corresponding comparison in H3K27ac data, we looked at the distribution of absolute fold-change for regions with significant difference in H3K27ac (with significance defined by diffBind, as explained previously). This analysis showed that while H3K27ac change is highly (and directionally) affected by EWS-FLI1 binding, DNA methylation change is not.

Supplemental References

Assenov, Y., Müller, F., Lutsik, P., Walter, J., Lengauer, T., and Bock, C. (2014). Comprehensive analysis of DNA methylation data with RnBeads. Nat Methods *11*, 1138-1140.

Ban, J., Bennani-Baiti, I.M., Kauer, M., Schaefer, K.L., Poremba, C., Jug, G., Schwentner, R., Smrzka, O., Muehlbacher, K., Aryee, D.N.*, et al.* (2008). EWS-FLI1 suppresses NOTCH-activated p53 in Ewing's sarcoma. Cancer Res *68*, 7100-7109.

Bock, C. (2012). Analysing and interpreting DNA methylation data. Nat Reviews Genet *13*, 705-719.

Bolger, A.M., Lohse, M., and Usadel, B. (2014). Trimmomatic: a flexible trimmer for Illumina sequence data. Bioinformatics *30*, 2114-2120.

Buenrostro, J.D., Giresi, P.G., Zaba, L.C., Chang, H.Y., and Greenleaf, W.J. (2013). Transposition of native chromatin for fast and sensitive epigenomic profiling of open chromatin, DNA-binding proteins and nucleosome position. Nat Methods *10*, 1213-1218.

Calo, E., and Wysocka, J. (2013). Modification of enhancer chromatin: what, how, and why? Mol Cell *49*, 825-837.

Carrillo, J., Garcia-Aragoncillo, E., Azorin, D., Agra, N., Sastre, A., Gonzalez-Mediero, I., Garcia-Miguel, P., Pestana, A., Gallego, S., Segura, D.*, et al.* (2007). Cholecystokinin down-regulation by RNA interference impairs Ewing tumor growth. Clin Cancer Res *13*, 2429-2440.

ENCODE Project Consortium (2012). An integrated encyclopedia of DNA elements in the human genome. Nature *489*, 57-74.

Kim, D., Pertea, G., Trapnell, C., Pimentel, H., Kelley, R., and Salzberg, S.L. (2013). TopHat2: accurate alignment of transcriptomes in the presence of insertions, deletions and gene fusions. Genome Biol *14*, R36.

Krueger, F., and Andrews, S.R. (2011). Bismark: a flexible aligner and methylation caller for bisulfite-seq applications. Bioinformatics *27*, 1571-1572.

Langmead, B., Trapnell, C., Pop, M., and Salzberg, S.L. (2009). Ultrafast and memory-efficient alignment of short DNA sequences to the human genome. Genome Biol *10*, R25.

Liberzon, A., Subramanian, A., Pinchback, R., Thorvaldsdottir, H., Tamayo, P., and Mesirov, J.P. (2011). Molecular signatures database (MSigDB) 3.0. Bioinformatics *27*, 1739-1740.

Liu, T., Ortiz, J.A., Taing, L., Meyer, C.A., Lee, B., Zhang, Y., Shin, H., Wong, S.S., Ma, J., Lei, Y., *et al.* (2011). Cistrome: an integrative platform for transcriptional regulation studies. Genome Biol *12*, R83.

Liu, Y., Siegmund, K.D., Laird, P.W., and Berman, B.P. (2012). Bis-SNP: combined DNA methylation and SNP calling for Bisulfite-seq data. Genome Biol *13*, R61.

Loven, J., Hoke, H.A., Lin, C.Y., Lau, A., Orlando, D.A., Vakoc, C.R., Bradner, J.E., Lee, T.I., and Young, R.A. (2013). Selective inhibition of tumor oncogenes by disruption of super-enhancers. Cell *153*, 320-334.

Sheffield, N.C., and Furey, T.S. (2012). Identifying and characterizing regulatory sequences in the human genome with chromatin accessibility assays. Genes *3*, 651-670.

Sheffield, N.C., Thurman, R.E., Song, L., Safi, A., Stamatoyannopoulos, J.A., Lenhard, B., Crawford, G.E., and Furey, T.S. (2013). Patterns of regulatory activity across diverse human cell types predict tissue identity, transcription factor binding, and long-range interactions. Genome Res *23*, 777-788.

Trapnell, C., Hendrickson, D.G., Sauvageau, M., Goff, L., Rinn, J.L., and Pachter, L. (2013). Differential analysis of gene regulation at transcript resolution with RNA-seq. Nat Biotechnol *31*, 46-53.

Whyte, W.A., Orlando, D.A., Hnisz, D., Abraham, B.J., Lin, C.Y., Kagey, M.H., Rahl, P.B., Lee, T.I., and Young, R.A. (2013). Master transcription factors and mediator establish super-enhancers at key cell identity genes. Cell *153*, 307-319.

Xi, Y., Bock, C., Müller, F., Sun, D., Meissner, A., and Li, W. (2012). RRBSMAP: a fast, accurate and user-friendly alignment tool for reduced representation bisulfite sequencing. Bioinformatics *28*, 430-432.

Zhang, Y., Liu, T., Meyer, C.A., Eeckhoute, J., Johnson, D.S., Bernstein, B.E., Nusbaum, C., Myers, R.M., Brown, M., Li, W., *et al.* (2008). Model-based analysis of ChIP-Seq (MACS). Genome Biol *9*, R137.
