# Supplementary material for: Epigenome Mapping Reveals Distinct Modes of Gene Regulation and Widespread Enhancer Reprogramming by the Oncogenic Fusion Protein EWS-FLI1
Source: Cell Rep. 2015 Feb 19;10(7):1082–95. doi: 10.1016/j.celrep.2015.01.042 (PMC4542316; doi:10.1016/j.celrep.2015.01.042)
Supplement: Document S2. Article plus Supplemental Information [file mmc3.pdf]

Cell Reports

Article

# Epigenome Mapping Reveals Distinct Modes of Gene Regulation and Widespread Enhancer Reprogramming by the Oncogenic Fusion Protein EWS-FLI1

## Graphical Abstract

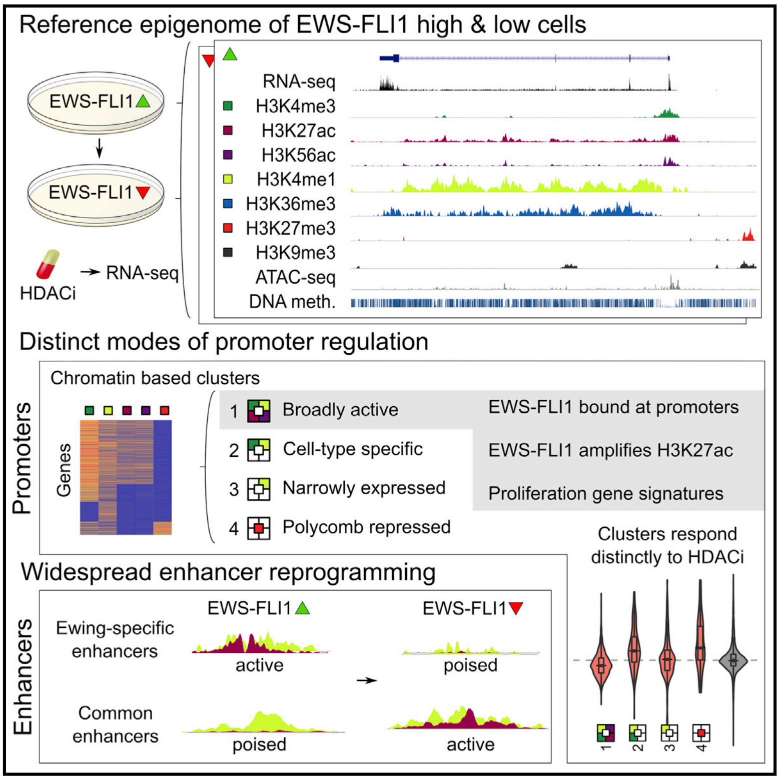


## Highlights

- Reference epigenome maps identify widespread epigenetic change in Ewing sarcoma cells
- EWS-FLI1-regulated genes fall into clusters with characteristic chromatin signatures
- Transcriptome response to HDAC inhibitors depends on promoter-specific histone marks
- EWS-FLI1 induces global changes in H3K27ac and genome-wide enhancer reprogramming

## Authors

Eleni M. Tomazou, Nathan C. Sheffield, ..., Christoph Bock, Heinrich Kovar

## Correspondence

cbock@cemm.oeaw.ac.at (C.B.),
heinrich.kovar@ccri.at (H.K.)

## In Brief

EWS-FLI1 is an oncogenic fusion protein and the main driver of Ewing sarcoma. Tomazou et al. establish comprehensive epigenome maps for an EWS-FLI1-dependent cell line. Based on these data, they identify clusters of epigenetically regulated genes and a unique enhancer signature that is associated with EWS-FLI1 oncogene addiction.

Tomazou et al., 2015, Cell Reports *10*, 1082–1095
February 24, 2015 ©2015 The Authors
http://dx.doi.org/10.1016/j.celrep.2015.01.042

OPEN ACCESS
CellPress

Cell Reports
Article

# Epigenome Mapping Reveals Distinct Modes of Gene Regulation and Widespread Enhancer Reprogramming by the Oncogenic Fusion Protein EWS-FLI1

Eleni M. Tomazou,[1,6] Nathan C. Sheffield,[2,6] Christian Schmidl,[2] Michael Schuster,[2] Andreas Schönegger,[2] Paul Datlinger,[2] Stefan Kubicek,[2] Christoph Bock,[2,3,4,*] and Heinrich Kovar[1,5,*]
[1]Children's Cancer Research Institute, St. Anna Kinderkrebsforschung, 1090 Vienna, Austria
[2]CeMM Research Center for Molecular Medicine of the Austrian Academy of Sciences, 1090 Vienna, Austria
[3]Department of Laboratory Medicine, Medical University of Vienna, 1090 Vienna, Austria
[4]Max Planck Institute for Informatics, 66123 Saarbrücken, Germany
[5]Department of Pediatrics, Medical University of Vienna, 1090 Vienna, Austria
[6]Co-first author
*Correspondence: cbock@cemm.oeaw.ac.at (C.B.), heinrich.kovar@ccri.at (H.K.)
http://dx.doi.org/10.1016/j.celrep.2015.01.042
This is an open access article under the CC BY license (http://creativecommons.org/licenses/by/4.0/).

## SUMMARY

**Transcription factor fusion proteins can transform cells by inducing global changes of the transcriptome, often creating a state of oncogene addiction. Here, we investigate the role of epigenetic mechanisms in this process, focusing on Ewing sarcoma cells that are dependent on the EWS-FLI1 fusion protein. We established reference epigenome maps comprising DNA methylation, seven histone marks, open chromatin states, and RNA levels, and we analyzed the epigenome dynamics upon downregulation of the driving oncogene. Reduced EWS-FLI1 expression led to widespread epigenetic changes in promoters, enhancers, and super-enhancers, and we identified histone H3K27 acetylation as the most strongly affected mark. Clustering of epigenetic promoter signatures defined classes of EWS-FLI1-regulated genes that responded differently to low-dose treatment with histone deacetylase inhibitors. Furthermore, we observed strong and opposing enrichment patterns for E2F and AP-1 among EWS-FLI1-correlated and anticorrelated genes. Our data describe extensive genome-wide rewiring of epigenetic cell states driven by an oncogenic fusion protein.**

## INTRODUCTION

Fusion proteins are a common cause of cancer. They often establish a state of oncogene addiction that makes cancer cells vulnerable to losing the fusion protein's biological function (Macconaill and Garraway, 2010). Direct pharmacological inhibition has been highly effective for certain kinase fusion proteins but is difficult for transcription factor fusions (Mitelman et al., 2004). It is therefore important to understand the regulatory mechanisms in fusion-protein-driven cancers in order to identify indirect ways of interfering with these oncogenes.

Here, we focus on epigenetic deregulation as a mechanism by which an oncogenic fusion protein may rewire cells for malignancy (Chen et al., 2010). We mapped the genome-wide dynamics of chromatin marks in a cellular model that is dependent on the EWS-FLI1 fusion protein. EWS-FLI1 is the most common initiating event in Ewing sarcoma, a pediatric cancer for which few therapeutic options exist (Bernstein et al., 2006). This oncogenic fusion protein originates from a chromosomal translocation that fuses the activator domain of the RNA binding protein EWS to the DNA binding domain of the FLI1 transcription factor (Delattre et al., 1994), and its expression results in upregulation and downregulation of several hundred target genes (Kovar, 2010). Ewing sarcoma has a lower rate of somatic mutations than most cancers (Brohl et al., 2014; Crompton et al., 2014; Huether et al., 2014; Lawrence et al., 2013; Tirode et al., 2014), suggesting that EWS-FLI1-driven tumors may be particularly dependent on deregulation of the epigenome.

To study EWS-FLI1-associated epigenetic changes, we performed comprehensive epigenome mapping in Ewing-sarcoma-derived cells, following the standards of the International Human Epigenome Consortium (http://ihec-epigenomes.net). Integrative bioinformatic analysis identified significant associations between EWS-FLI1 binding and the chromatin states of promoters, enhancers, and super-enhancers. We also compared the epigenomes before and after knockdown of the EWS-FLI1 fusion protein, allowing us to define clusters of EWS-FLI1-regulated genes. We validated the relevance of our gene clustering by measuring the transcriptome response to epigenome-modulating drugs, and we identified EWS-FLI1-specific enhancer and super-enhancer signatures that are dependent on EWS-FLI1 expression.

Our results highlight the prevalence, complexity, and dynamics of epigenome and transcriptome rewiring orchestrated by EWS-FLI1, and they provide initial insights into the role of the epigenome in solid cancer cells that depend on an oncogenic fusion protein.

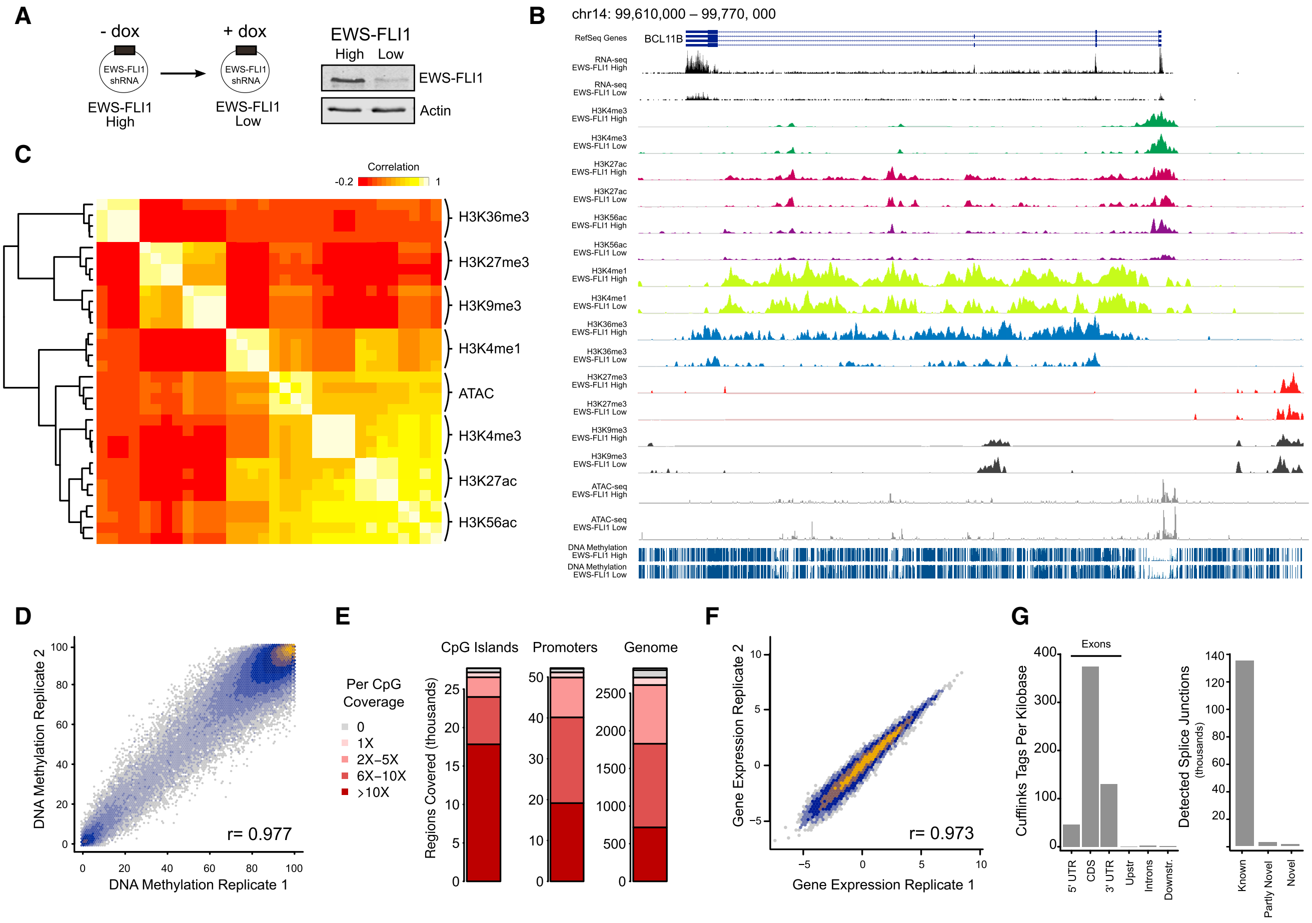


**Figure 1. Reference Epigenome Maps of Ewing-Sarcoma-Derived Cells at High and Low Levels of EWS-FLI1 Expression**

(A) Schematic of an inducible small hairpin RNA (shRNA) system in the Ewing sarcoma cell line A673, which allows for efficient switching between high and low EWS-FLI1 expression levels. A representative western blot illustrates the efficiency of induced EWS-FLI1 knockdown.

(B) Genome browser screenshot of the reference epigenome maps at a known EWS-FLI1 target gene (*BCL11B*), shown for high and low levels of EWS-FLI1 expression. The tracks visualize RNA-seq data, ChIP-seq for seven histone marks, DNA methylation levels at single-CpG resolution, and ATAC-seq signal as a measure of open chromatin. All data are publicly available for interactive exploration and download (http://tomazou2015.computational-epigenetics.org).

(C) Heatmap showing the genome-wide correlation of ChIP-seq and ATAC-seq signals in 1-kb tiling windows. Light colors (white/yellow) correspond to strong correlation and dark colors (red) correspond to weak or negative correlation.

(D) Scatterplot showing the genome-wide correlation of average DNA methylation levels (1-kb tiling windows) between biological replicates in the EWS-FLI1-high state.

(E) Bar charts showing DNA methylation coverage for combined WGBS and RRBS data in CpG islands, promoter regions (1-kb region upstream of TSS), and 1-kb tiling windows across the genome.

(F) Scatterplot showing the genome-wide correlation of RNA levels (log FPKM [fragments per kilobase of transcript per million mapped reads] values for assembled transcripts) between biological replicates in the EWS-FLI1-high state.

(G) Bar charts showing RNA-seq coverage at different types of genomic regions and detection levels for known and novel splicing junctions.

See also Figure S1.

## RESULTS

### Reference Epigenome Mapping in a Cellular Model of EWS-FLI1 Dependence

Epigenome mapping is a powerful method for cataloging functional elements throughout the genome (Bernstein et al., 2012), and it can provide insights into the regulatory mechanisms that underlie changes of cell state (Rivera and Ren, 2013). To investigate the effect of EWS-FLI1 expression on epigenetic cell states, we mapped the epigenome of an Ewing sarcoma cell line (A673) that has emerged as a standard model for systems biology in Ewing sarcoma (http://www.ucd.ie/sbi/asset). This cell line is EWS-FLI1 dependent and was previously engineered to harbor a doxycycline-inducible small hairpin RNA against EWS-FLI1 (Carrillo et al., 2007). These cells retain a low level of EWS-FLI1 expression after knockdown, thus allowing us to compare, in an isogenic setting, the epigenomes of EWS-FLI1-high and EWS-FLI1-low cell states without causing extensive cell death (Figure 1A).

We established reference epigenome maps comprising DNA methylation, seven histone marks (H3K4me3, H3K4me1,

H3K27ac, H3K56ac, H3K36me, H3K27me3, and H3K9me3), open chromatin states, and RNA levels. DNA methylation was mapped both by whole-genome bisulfite sequencing (WGBS) and reduced representation bisulfite sequencing (RRBS), histone marks by chromatin immunoprecipitation sequencing (ChIP-seq), open chromatin states by assay for transposase-accessible chromatin with high-throughput sequencing (ATAC-seq), and RNA levels by strand-specific RNA sequencing (RNA-seq). Each experiment was performed in two biological replicates each for both the EWS-FLI1-high state (cells in normal growth conditions) and the EWS-FLI1-low state (53 hr after adding doxycycline to induce knockdown of EWS-FLI1). We also performed RNA-seq in cells that were treated with each of three histone deacetylase inhibitors to further dissect the relevance of histone acetylation in our model. In total, we generated 61 datasets and sequenced 2.9 billion reads comprising 221 billion base pairs (Table S1).

To facilitate data access and reuse by other researchers, we developed a website for online exploration and data download (http://tomazou2015.computational-epigenetics.org). Our dataset can be used to view and investigate the epigenetic changes in EWS-FLI1-dependent cells one gene at a time (Figure 1B) or to study patterns of epigenetic and transcriptional deregulation on a more global level, for example by annotating known and novel non-coding transcripts and alternative promoters that are EWS-FLI1 dependent (Figure S1).

Hierarchical clustering of the ChIP-seq and ATAC-seq data showed high consistency between experiments of the same type (Figure 1C). We also observed the expected separation into repressive histone marks (H3K9me3 and H3K27me3), transcription-associated histone marks (H3K36me3), and open-chromatin-associated marks (H3K4me3, H3K4me1, H3K27ac, H3K56ac, and ATAC-seq signal). For the DNA methylation data, which were combined from WGBS and RRBS experiments, we observed high correlation between replicates (Figure 1D) and broad coverage throughout the genome (Figure 1E). The correlation among biological replicates was equally high for the RNA-seq data (Figure 1F), and we were able to identify a small number of transcripts and splicing isoforms that appear to be specific to this cell type (Figures 1G and S1). In aggregate, these observations confirm the high technical data quality of the reference epigenome maps that we generated.

## Significant Association of Gene Expression, EWS-FLI1 Binding, and Open Chromatin

EWS-FLI1 is known to act as a transcriptional activator (Bailly et al., 1994; Ohno et al., 1993), but it also represses genes—indirectly by activating transcriptional repressor genes and potentially also directly by recruiting repressive protein complexes (Hahm et al., 1999; Prieur et al., 2004; Sankar et al., 2013). To investigate the genome-wide association among gene expression, EWS-FLI1 binding, and chromatin states, we stratified all genes by their RNA levels (Figure 2A, top) and by the distance from the transcription start site (TSS) to the nearest EWS-FLI1 binding peaks (Figure 2A, bottom), which were mapped previously (Bilke et al., 2013), and we plotted average ChIP-seq intensities for each histone mark around the TSS (Figures 2A and S2). Promoters of highly expressed genes and those bound by EWS-FLI1 had high levels of the open-chromatin-associated marks H3K4me3, H3K27ac, and H3K56ac. These marks also showed the characteristic dip at the TSS that is indicative of nucleosome-free regions (ENCODE Project Consortium, 2012), and they overlapped with a peak of open chromatin in the ATAC-seq data. H3K4me1 was similarly enriched in the wider promoter region but depleted in immediate vicinity of the TSS, reflecting its mutual exclusivity with H3K4me3. Furthermore, H3K36me3 levels were high in genic regions directly downstream of the TSS of genes that were highly expressed and bound by EWS-FLI1, whereas the repressive histone marks H3K9me3 and H3K27me3 were depleted in the promoters of these genes (Figures 2A and S2). Promoters with distal EWS-FLI binding (5–40 kb from the TSS) were no more enriched for the open-chromatin marks H3K4me3, H3K27ac, and H3K56ac than those showing no EWS-FLI1 binding within 40 kb from the TSS, but they were more strongly depleted for the repressive-histone marks H3K27me3 and H3K9me3.

Combining gene expression data with EWS-FLI1 binding, we observed that proximal and distal binding of EWS-FLI1 were both associated with higher RNA levels (Figure 2B), suggesting that EWS-FLI1 acts as a transcriptional activator not only when it binds in direct vicinity to the TSS but also at enhancers outside of the promoter region. Proximal binding sites of EWS-FLI1 carried the characteristic histone patterns of promoter regions (Figure 2C), whereas distal EWS-FLI1 binding sites showed the chromatin signature of active enhancers with high levels of H3K27ac and H3K4me1, low levels of H3K4me3, and a dip in H3K27ac levels at the binding site (Figure 2D). H3K27ac was consistently associated with EWS-FLI1 binding, both for promoter regions and for distal enhancer elements. To identify DNA methylation patterns associated with EWS-FLI1 binding, we thus compared DNA methylation levels of H3K27ac peaks with and without EWS-FLI1 binding, and we observed significantly lower DNA methylation levels among the EWS-FLI1-bound regions (Figure 2E). This result is consistent with recent evidence that active transcription factors can reduce DNA methylation at their binding sites (Stadler et al., 2011).

Collectively, our results support a strong association of EWS-FLI1 binding with high gene expression, open chromatin, nucleosome depletion, and reduced DNA methylation. These patterns hold true for both promoters and enhancers and for both proximal and distal binding. We did not observe widespread co-localization of EWS-FLI1 with repressive chromatin, suggesting that the repressive effect of EWS-FLI1 observed for a sizable fraction of its target genes (Figures 3A and 3B) is either indirect or caused by the depletion of active histone marks.

## Chromatin-Based Clusters of EWS-FLI1-Dependent Transcripts

Several studies mapped EWS-FLI1 target genes using expression microarrays and/or ChIP-seq analysis of EWS-FLI1 binding (Bilke et al., 2013; Kauer et al., 2009; Prieur et al., 2004; Riggi et al., 2008; Smith et al., 2006; Tirode et al., 2007). Both approaches are useful but limited in their insight into the underlying regulatory mechanisms. We hypothesized that epigenome maps could help identify distinct modes of transcriptional regulation, and we devised a bioinformatic approach to classify

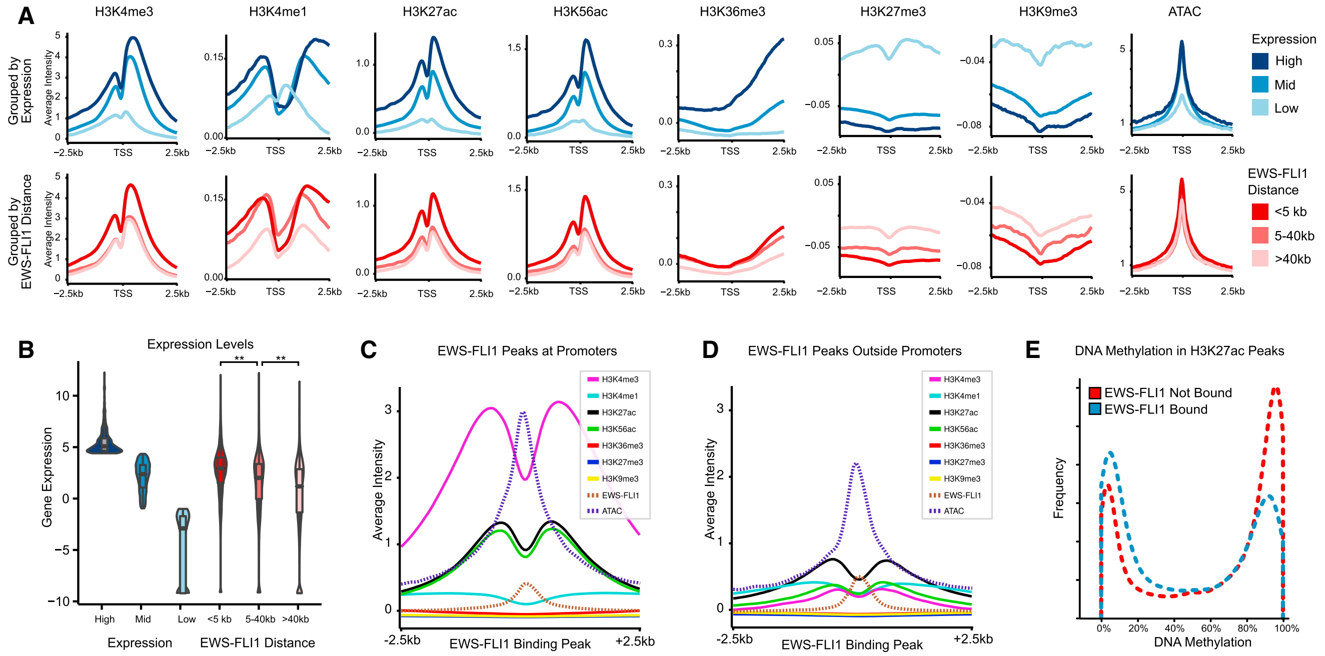

**Figure 2. Genome-wide Association of Chromatin State with RNA Levels and EWS-FLI1 Binding**

(A) Composite plots showing average ChIP-seq and ATAC-seq intensities around the TSS. Gene promoters are stratified in two ways: by RNA levels (top row, shades of blue) and by their distance to the nearest EWS-FLI1 binding peak (bottom row, shades of red).

(B) Violin plot visualizing the distribution of RNA levels for highly expressed genes (top 20%), moderately expressed genes (20% to 80%), and lowly expressed genes (bottom 10%) and for genes with EWS-FLI1 binding peaks located in the promoter region (<5 kb from the TSS), distal to the TSS (5–40 kb), or nowhere nearby (>40 kb). The observed expression differences based on distance to nearest EWS-FLI1 peaks were highly significant ($p < 10^{-100}$, Wilcoxon signed-rank test).

(C) Composite plots showing the average ChIP-seq and ATAC-seq intensities for EWS-FLI1 binding peaks located proximal to the nearest TSS (<5 kb from the TSS). All ChIP-seq data are on the same scale, while the ATAC-seq intensities were rescaled to fit the plot.

(D) Composite plots for EWS-FLI1 binding peaks located distal to the nearest TSS (>5 kb from the TSS). The scale is identical to (C).

(E) Histogram showing the distribution of DNA methylation levels in the EWS-FLI1-high state among H3K27ac peaks that are EWS-FLI1 bound (blue) versus not bound (red). The two distributions are significantly different ($p < 10^{-16}$, Kolmogorov-Smirnov test).

See also Figure S2.

genes in a way that takes biological knowledge about chromatin regulation into account (Figure S3; Supplemental Experimental Procedures).

First, we identified all genes that were significantly downregulated or upregulated upon EWS-FLI1 knockdown, according to our RNA-seq data (Figure 3A). To emphasize that the regulatory relationship with EWS-FLI1 may be direct or indirect, we refer to these genes as "EWS-FLI correlated" and "EWS-FLI1 anticorrelated" rather than "EWS-FLI1 activated" and "EWS-FLI1 repressed." In total, 1,287 transcripts were significantly correlated with EWS-FLI1 (i.e., more highly expressed in the EWS-FL1-high state than in the EWS-FLI1-low state, which is equivalent to downregulation upon EWS-FLI1 knockdown), and 1,446 transcripts were significantly anticorrelated (i.e., more highly expressed in the EWS-FLI1-low state, which is equivalent to upregulation upon EWS-FLI1 knockdown) (Figure 3B).

In a second step, we annotated all correlated and anticorrelated transcripts with ChIP-seq intensity levels in their promoter regions, focusing on the five histone marks that are generally associated with gene promoters (Figure 3C). We then clustered the transcripts based on their histone marks in the state where they are more lowly expressed (correlated transcripts: EWS-FLI1-low state after EWS-FLI1 knockdown; anticorrelated transcripts: EWS-FLI1-high state before EWS-FLI1 knockdown). To that end, an initial step of unsupervised clustering was followed by expert classification of selected transcripts and supervised prediction of cluster membership for all transcripts (Figure S3). Cluster 1 comprises transcripts carrying all four active-promoter marks (H3K4me3, H3K4me1, H3K27ac, and H3K56ac), but not the repressive H3K27me3 mark; cluster 2 transcripts carry both H3K4me3 and H3K4me1 but lack the histone-acetylation marks; cluster 3 transcripts are marked by H3K4me1 only; and cluster 4 is characterized by the repressive mark H3K27me3 in the presence or absence of H3K4 methylation. Using a logistic regression model for predicting cluster membership, most correlated and anticorrelated transcripts (82% and 80%) were placed unambiguously in one of the four clusters (Figures 3D and 3E). Examples of EWS-FLI1-anticorrelated transcripts for each of the four clusters are shown in Figures 3F–3I.

## Functional Characteristics and Chromatin Dynamics of EWS-FLI1-Regulated Transcripts

To validate the biological relevance of our chromatin-based gene clusters, we analyzed gene set enrichment and overlap of their

OPEN ACCESS
CellPress

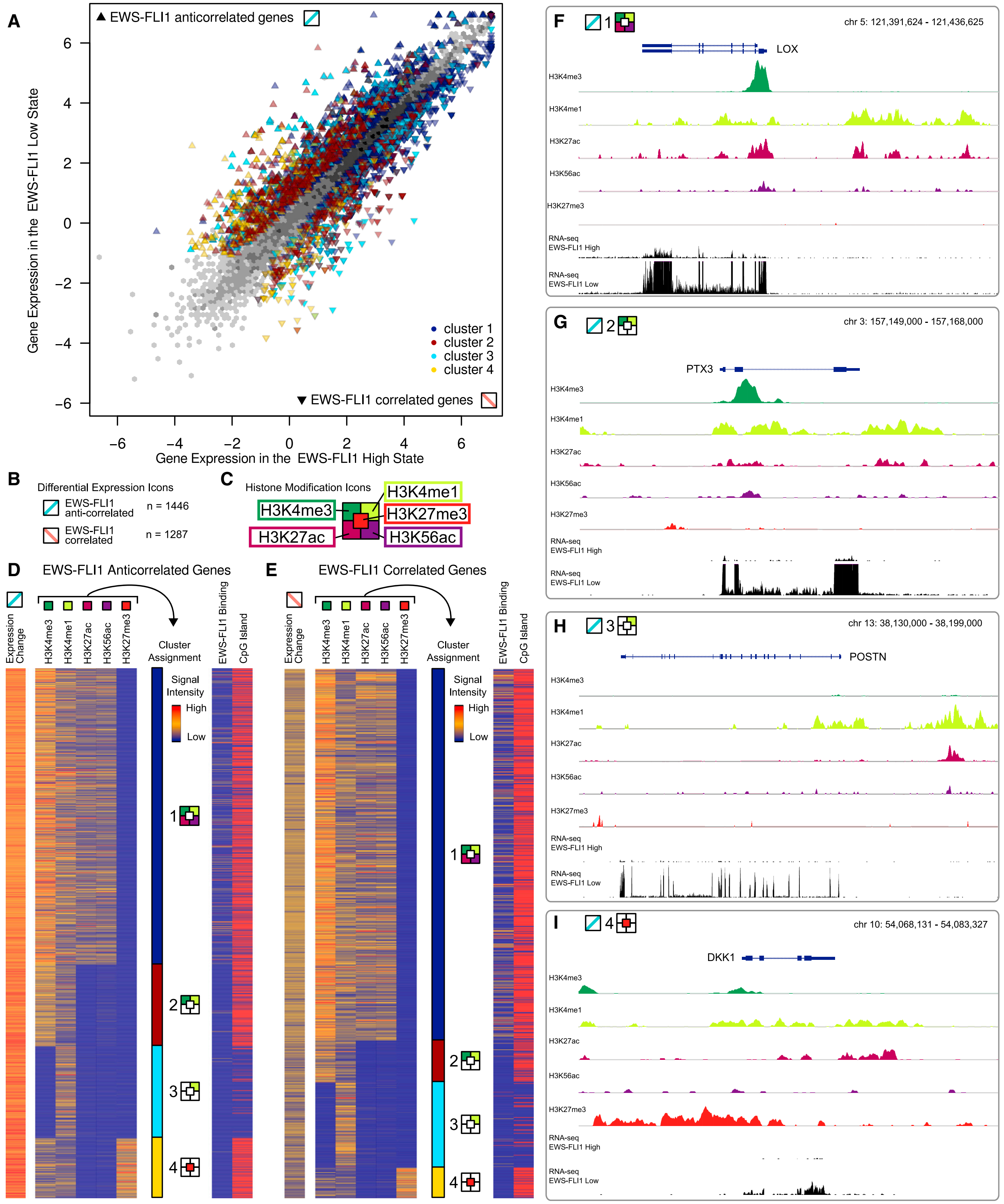


*(legend on next page)*

promoters with catalogs of regulatory elements. Each cluster showed specific enrichment patterns, which were similar among EWS-FLI1-correlated and anticorrelated transcripts. Cluster 1 promoters were highly enriched for polymerase binding and transcription initiation activity in a broad range of cell types (Figure 4A), and they were also enriched for CpG islands (Figures 3D and 3E), which tend to associate with strong and widely active promoters. Cluster 2 promoters had lower overlap with CpG islands and a strong association with tissue-specific patterns of open versus repressed chromatin and with binding of tissue-specific transcription factors. Cluster 3 comprised tissue-specific genes that lack CpG islands. Finally, cluster 4 transcripts were strongly enriched for Polycomb repressive complex 2 binding across many cell types.

In addition to the enrichment patterns that were shared between EWS-FLI1-correlated and anticorrelated clusters, we also observed interesting differences. For example, EWS-FLI1 promoter binding was more common for EWS-FLI1-correlated transcripts (i.e., those that are downregulated after knockdown of EWS-FLI1) than for anticorrelated transcripts (Figures 3D, 3E, and 4A). Furthermore, when comparing each EWS-FLI1-correlated cluster to the corresponding EWS-FLI1-anticorrelated cluster (Figure 4B), we observed a striking enrichment for binding of E2F transcription factors among the EWS-FLI1-correlated transcripts, reinforcing a previously reported link between EWS-FLI1 and E2F (Bilke et al., 2013). The EWS-FLI1-correlated transcripts were also enriched for proliferation gene sets, consistent with the ability of EWS-FLI1 to accelerate cell growth. In contrast, anticorrelated transcripts were enriched for gene sets that are characteristic of adult stem cells and for binding of both components of the AP-1 transcription factor, FOS and JUN.

The chromatin-based gene clusters not only showed distinct patters of functional enrichment but also responded differently to EWS-FLI1 knockdown (Figure 4C). Among the EWS-FLI1-correlated transcripts, H3K27ac was most strongly reduced at cluster 1 promoters after EWS-FLI1 knockdown, whereas H3K4me3 and H3K4me1 signals were largely retained even in the EWS-FLI1-low state. In contrast, clusters 2 and 3 showed reduced levels for all three marks (H3K4me3, H3K4me1, and H3K27ac), and cluster 4 promoters underwent relatively minor changes upon EWS-FLI1 knockdown. The EWS-FLI1-anticorrelated clusters showed a more uniform response across clusters with increases in H3K4me3 and H3K27ac (Figure 4D). Together, these observations indicate that EWS-FLI1 expression promotes transcription of its target genes in at least two distinct ways: first, by further increasing H3K27ac levels and transcription of genes that are already widely expressed in proliferating cells (correlated cluster 1); and second, by establishing or maintaining the chromatin signature of active promoters (H3K4me3, H3K4me1, and H3K27ac) at cell type specific genes that would otherwise be silent in these cells (correlated clusters 2 and 3). Furthermore, the relatively uniform response among the anticorrelated clusters indicates that EWS-FLI1 mediated repression involves mechanisms that decrease H3K27ac in similar ways across all four EWS-FLI1 anticorrelated clusters.

Based on these promoter-centric analyses, H3K27ac and, to a lesser degree, H3K4me3 and H3K4me1 emerged as the histone marks that were most strongly affected by EWS-FLI1 knockdown. This result was corroborated by western blots for all seven histone marks in additional Ewing sarcoma cell lines (TC252, SK-N-MC, and ST-ET-7.2), where we observed a global reduction in H3K4me3 and H3K27ac levels upon knockdown of EWS-FLI1 (Figure 4E). These data indicate that the global and EWS-FLI1-dependent increase of active chromatin may represent a key mechanism of transcriptional rewiring in Ewing sarcoma cells.

## Cluster-Specific Transcriptional Response to Treatment with Histone Deacetylase Inhibitors

Given that H3K27ac was most variable between the chromatin-based gene clusters and also most dynamic upon EWS-FLI1 knockdown (Figures 3 and 4), we hypothesized that the clustered genes should respond differently to induced global changes of acetylation, which would support the clusters' usefulness for identifying distinct modes of gene regulation among EWS-FLI1 target genes. To test this hypothesis, we treated the cells with histone acetyltransferase (HAT) inhibitors and histone deacetylase (HDAC) inhibitors. These experiments were done for both the EWS-FLI1-high and the EWS-FLI1-low cell state, and they used drug concentrations that were low enough to avoid broad toxicity. None of the three HAT inhibitors that we tested (C646, CPTH2, and anacardic acid) had an effect on cell survival or gene expression at the chosen concentrations (data not shown); hence, we focused our analysis primarily on the HDAC inhibitors (Figures 5 and S4).

HDAC inhibitors are being evaluated as drug candidates for Ewing sarcoma (Arnaldez and Helman, 2014), but here we used them solely as a chemical biology tool for validating our gene clustering, and at much lower concentrations than if our goal were to selectively kill EWS-FLI1-expressing cells. For two out of three HDAC inhibitors tested—namely, romidepsin

**Figure 3. Four Chromatin-Based Clusters of EWS-FLI1-Regulated Genes**

(A) Scatterplot comparing RNA levels between the EWS-FLI1-high state (x axis, RNA collected prior to knockdown of EWS-FLI1) and the EWS-FLI1-low state (y axis, RNA collected 53 hr after inducing shRNA knockdown). Differentially expressed genes are indicated by triangles colored according to the gene cluster they belong to (as defined in D and E).

(B) Total number of differentially expressed genes and icons depicting their change as EWS-FLI1 correlated (red) or EWS-FLI1 anticorrelated (blue). This graphical coding is used throughout the figures to indicate which epigenetic and transcriptional changes move in the same or the opposite direction compared to the EWS-FLI1 levels upon knockdown.

(C) Promoter-associated histone marks that were included in the semi-supervised clustering of EWS-FLI1-correlated and anticorrelated transcripts. Variations of this icon are used to depict each of the four chromatin-based gene clusters, with colored fields indicating the presence of the corresponding histone mark among the clustered gene promoters.

(D and E) Heatmaps and chromatin-based clustering of EWS-FLI1-anticorrelated genes (D) and EWS-FLI-correlated genes (E).

(F–I) Genome browser screenshots with one example gene from each anticorrelated cluster. Histone data are shown for the EWS-FLI1-high state.

See also Figure S3.

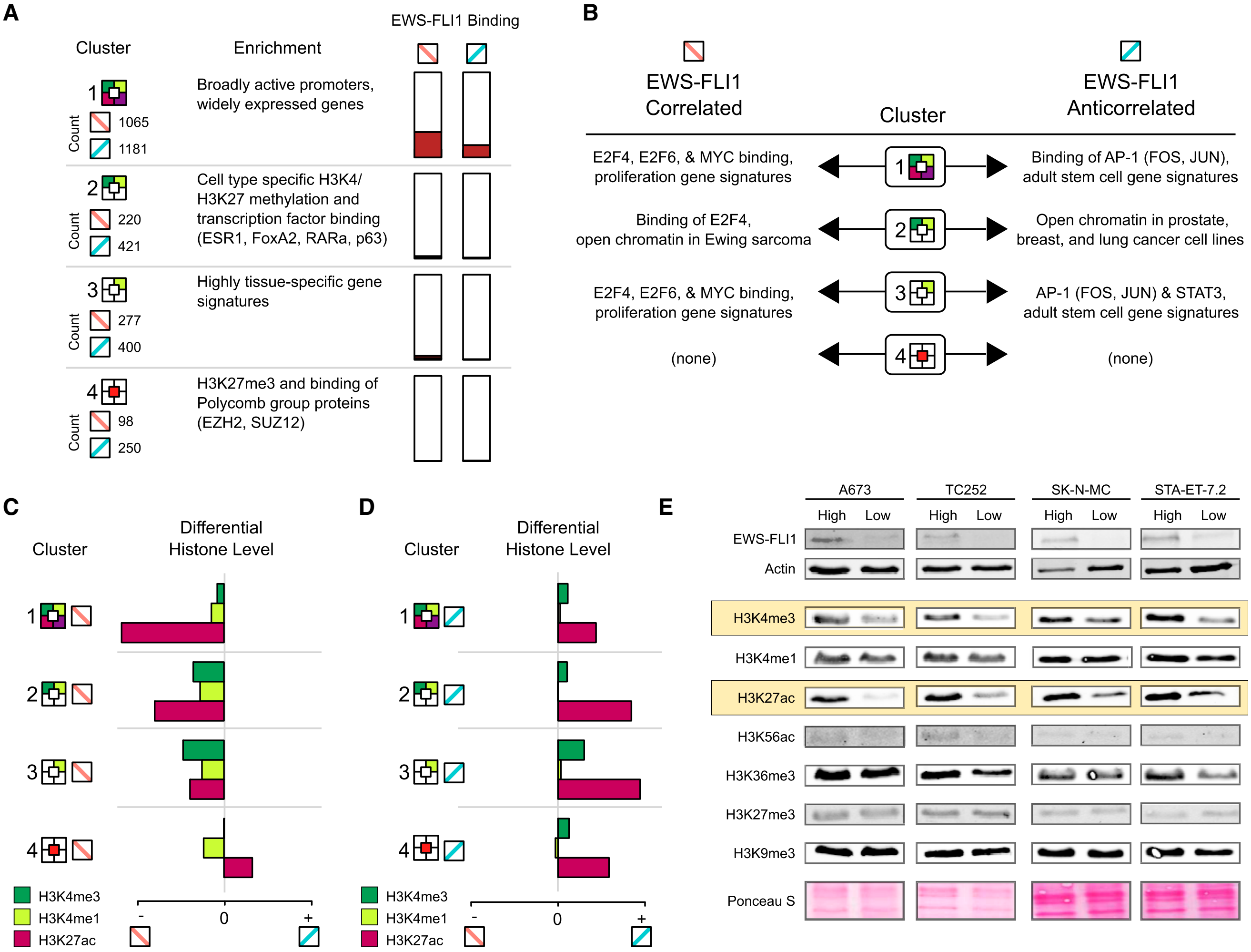


**Figure 4. Functional Enrichment and Changes in Histone Marks among Chromatin-Based Gene Clusters**

(A) Summary statistics and functional enrichment analysis for the chromatin-based gene clusters (see Figure 3B and 3C for an explanation of the colored icons). The enrichment column summarizes the most significant terms when comparing each cluster to all other clusters (see http://tomazou2015.computational-epigenetics.org for tables with all enriched terms).

(B) Functional enrichment analysis of correlated versus anticorrelated genes within each cluster. The enrichment columns summarize the most significant terms when comparing each cluster of EWS-FLI1-correlated genes to the corresponding cluster of EWS-FLI1-anticorrelated genes.

(C and D) Bar charts summarizing the changes in histone marks at promoters of correlated (C) and anticorrelated (D) genes in response to EWS-FLI1 knockdown. The scale of the x axis is the same across clusters and panels, and it indicates relative increase (+) or decrease (–) of the ChIP-seq signal intensity after EWS-FLI1 knockdown.

(E) Western blots showing global levels of EWS-FLI1 and of seven histone marks for the EWS-FLI1-high and EWS-FLI1-low states. Actin and Ponceau S staining were used as loading controls for EWS-FLI1 and histone marks, respectively. Data are shown for the inducible knockdown cell line (A673) and for three additional Ewing sarcoma cell lines (TC252, SK-N-MC, and STA-ET-7.2). Boxes highlight H3K27ac and H3K4me3 as the two histone marks whose global levels correlate with EWS-FLI1 expression in all four Ewing sarcoma cell lines. All western blots were done in multiple biological replicates, and this panel combines representative blots from these experiments.

(FK-228) and entinostat (MS-275), but not Vorinostat (SAHA)—we were able to identify concentrations that fulfilled all three criteria that were crucial for our experiment: (1) no effect on cell viability (Figures 5A and S4A), (2) global increase in histone acetylation levels (Figure 5B), and (3) no effect on EWS-FLI1 expression levels as a potential confounder (Figure 5B).

We performed RNA-seq to measure the transcriptome response to each HDAC inhibitor at the chosen concentrations (Figures 5C–5E, S4B, and S4C). Both romidepsin and entinostat specifically upregulated the expression of EWS-FLI1-anticorrelated transcripts in cells with high EWS-FLI1 levels, suggesting that the (direct or indirect) repressive effect of EWS-FLI1 is partially reversed by treating EWS-FLI1-high cells with HDAC inhibitors. Among the anticorrelated transcripts, cluster 1 was least affected by treatment with HDAC inhibitors, most likely because cluster 1 promoters already carried high levels of H3K27ac and may therefore be less sensitive to HDAC inhibition (Figures 5D and S4C). In contrast, clusters 2, 3, and 4 carried low

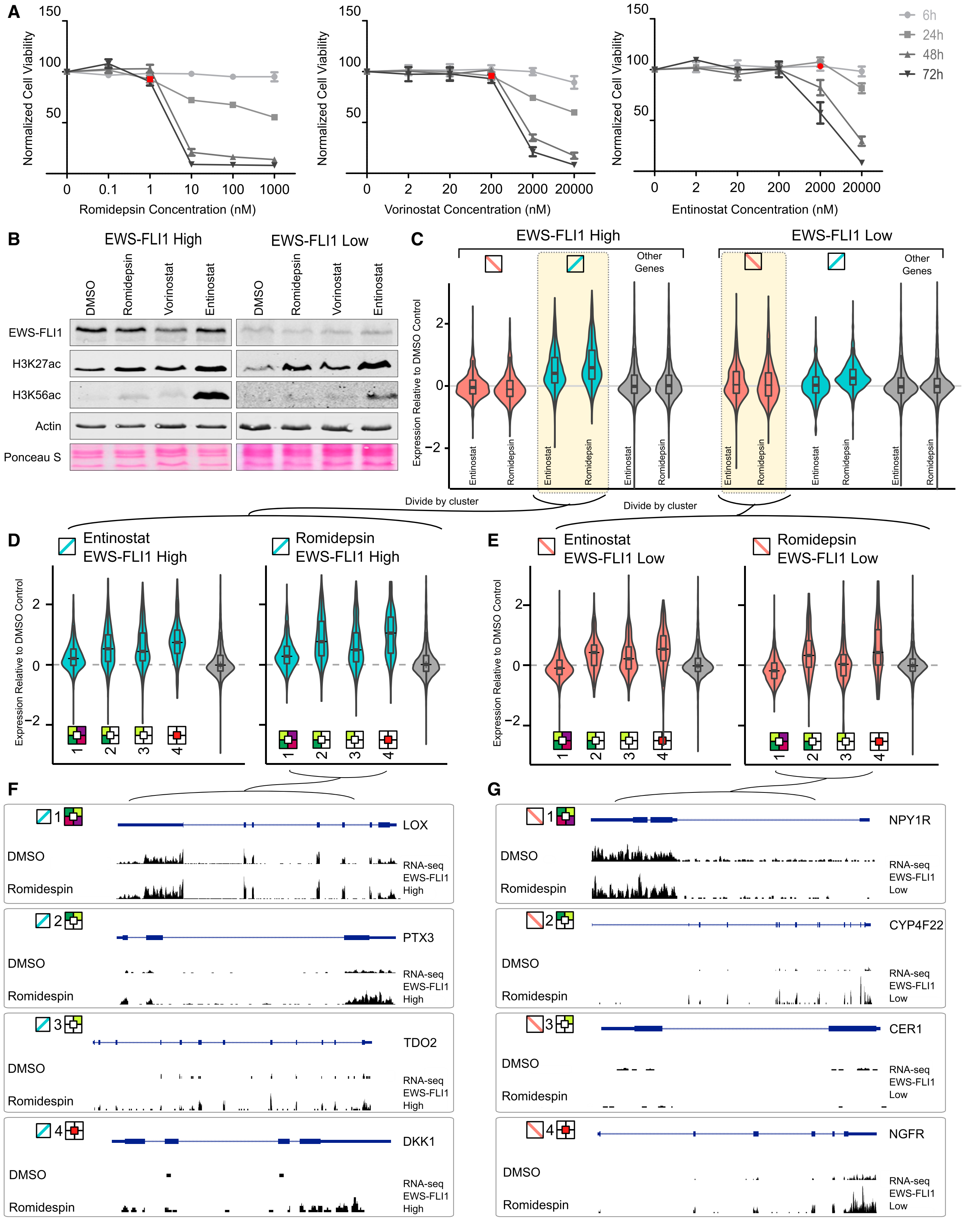


(legend on next page)

levels of H3K27ac in the EWS-FLI1-high state and responded more strongly to treatment with HDAC inhibitors. Examples for each of the four clusters are shown in Figure 5F.

Among the EWS-FLI1-correlated genes, there was no global trend in either direction (Figure 5C), but we observed strong cluster-specific differences in the EWS-FLI1-low state (Figures 5E and S4B). Clusters 2 and 4 were consistently upregulated in response to HDAC inhibition, whereas no such trend was observed for cluster 3, and cluster 1 genes were even slightly downregulated. Specific examples are shown in Figure 5G. These results indicate that EWS-FLI1 activates certain genes in cluster 2 and 4 by interfering with HDAC function, and treatment with HDAC inhibitors appears to recapitulate this effect in EWS-FLI1-low cells.

## Widespread Reprogramming of Enhancers and Super-Enhancers by EWS-FLI1

Our promoter-centric analysis (Figures 3 and 4) established H3K4me3 and H3K27ac as the two histone marks that were most strongly associated with EWS-FLI1 expression. H3K4me3 is a prototypic promoter mark, and the observed changes in H3K4me3 were indeed located almost exclusively in promoter regions. By contrast, not only does H3K27ac occur in promoter regions (where it tends to overlap with H3K4me3), but it is also a defining mark of active enhancers when occurring in the absence of H3K4me3 (Creyghton et al., 2010; Rada-Iglesias et al., 2011). We therefore investigated the effect of EWS-FLI1 expression on H3K27ac beyond gene promoters, comparing H3K27ac patterns before and after knockdown of EWS-FLI1 across the genome. In total, 15,300 H3K27ac peaks showed significantly lower intensity in EWS-FLI1-low cells (“EWS-FLI1 correlated peaks”), and 18,727 H3K27ac peaks showed significantly higher intensity (“EWS-FLI1 anticorrelated peaks”). 27% of correlated peaks and only 6% of anticorrelated peaks overlapped with promoter regions (Figures 6A and S5A), indicating that the majority of EWS-FLI1-associated H3K27ac peaks are located at enhancer elements outside of promoter regions.

We observed striking differences between EWS-FLI1-correlated and anticorrelated H3K27ac peaks, which became apparent when we stratified all peaks by the strength of H3K27ac signal difference upon EWS-FLI1 knockdown. Among EWS-FLI1-correlated enhancers, only 10% of the top 20% most differential H3K27ac peaks were located in promoters, whereas 62% of the bottom 20% peaks (which are still significantly different for H3K27ac between the EWS-FLI1-high and low states) were located in promoters (Figure 6B). No such differences were observed for EWS-FLI1-anticorrelated peaks, of which 5% and 7% were located in promoters. We also identified strong differences in EWS-FLI1 binding, with 77% of the top hundred EWS-FLI1-correlated H3K27ac peaks being EWS-FLI bound, while the percentage for EWS-FLI1 binding fell to 20% when calculated across all EWS-FLI1-correlated peaks and to only 1% for EWS-FLI1-anticorrelated peaks (Figure 6C). Finally, for the other histone marks tested we observed much lower overlap of EWS-FLI1 (Figure S5B), indicating that EWS-FLI1 primarily drives H3K27ac at selected promoters and enhancers throughout the genome.

Our observation that the strongest EWS-FLI1-correlated enhancers were qualitatively different from other H3K27ac peaks is reminiscent of super-enhancers (Hnisz et al., 2013; Lovén et al., 2013; Whyte et al., 2013). We therefore investigated whether super-enhancers occur in EWS-FLI1 expressing cells and how they might respond to EWS-FLI1 knockdown. Using a published method for H3K27ac-based annotation of super-enhancers (Lovén et al., 2013; Whyte et al., 2013), we identified 697 super-enhancers in the EWS-FLI1-high state and annotated them with their neighboring genes (http://tomazou2015.computational-epigenetics.org). Overall, more than 10% of H3K27ac peaks in EWS-FLI1-high cells were located in super-enhancers (Figure S5C), and super-enhancers had much higher cumulative H3K27ac enrichment than typical enhancers (Figure 6D). This strong enrichment of H3K27ac was largely due to the exceeding length of super-enhancers (median length of 27,818 base pairs as compared to 1,196 base pairs for typical enhancers; Figure S5D) and not due to higher H3K27ac signal intensity per base pair (Figure S5E). As illustrated in Figure 6E, super-enhancers were essentially agglomerates of co-located H3K27ac peaks over a range of several dozen kilobases, while typical enhancers rarely comprised more than two H3K27ac peaks and were typically shorter than 2 kb. Several of the top super-enhancers were located near genes with known roles in cell proliferation, differentiation, and apoptosis. We also observed widespread loss of H3K27ac upon knockdown of EWS-FLI1 for some super-enhancers (Figure 6E), while others displayed more localized changes affecting only those H3K27ac peaks

**Figure 5. Transcriptome Response upon Treatment with Histone Deacetylase Inhibitors**

(A) Dose-response curves showing cell viability for three histone deacetylase (HDAC) inhibitors at different concentrations and different time points. Data are shown for the EWS-FLI1-high state. Error bars represent the SEM of triplicate experiments, and red squares indicate well-tolerated concentrations that were selected for measuring the transcriptome response.

(B) Western blots showing the effect of three HDAC inhibitors on the global levels of histone acetylation and EWS-FLI1 expression in the EWS-FLI1-high state (left) and the EWS-FLI1-low state (right). Treatment with 0.1% DMSO was used as negative control for treatment effect, whereas actin and Ponceau S were included as loading controls for the western blot. All western blots were done in several biological replicates.

(C) Violin plots visualizing gene expression changes in response to treatment with sublethal doses of two HDAC inhibitors. Log fold change between treated and untreated samples (y axis) is shown for EWS-FLI1-correlated genes (red), EWS-FLI1-anticorrelated genes (blue) and all other genes (gray), separately for the EWS-FLI1-high state (left) and for the EWS-FLI1-low state (right). All values are means across two biological replicates.

(D and E) Violin plots visualizing gene expression changes for each of the four chromatin-based gene clusters (Figure 3), focusing on EWS-FLI-anticorrelated genes in the EWS-FLI1-high state (D) and on EWS-FLI-correlated genes in the EWS-FLI1-low state (E).

(F and G) Genome browser screenshots illustrating the RNA-seq response to romidepsin treatment. One example gene is shown for each gene cluster. Anticorrelated genes are shown in the EWS-FLI1-high state (F), and correlated genes are shown in the EWS-FLI1-low state (G).

See also Figure S4.

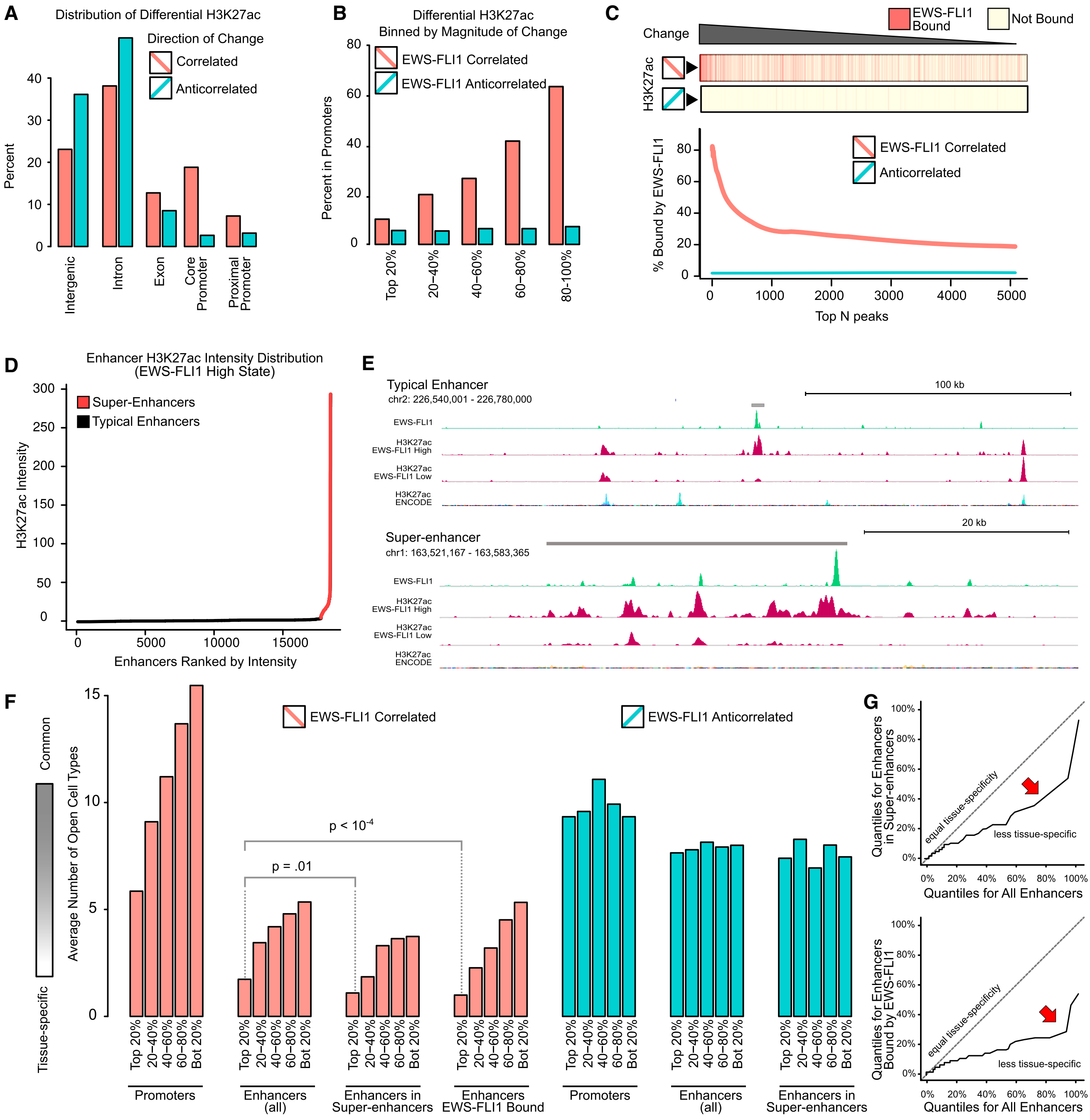


**Figure 6. Characteristic H3K27ac Patterns and Widespread Enhancer Reprogramming in EWS-FLI1-Dependent Cells**

(A) Differences in genomic localization between differential H3K27ac peaks that are correlated (higher intensity in the EWS-FLI1-high state) or anticorrelated (higher intensity in the EWS-FLI1-low state) with EWS-FLI1 levels.

(B) Bar charts showing the proportion of differential H3K27ac peaks that overlap with gene promoters (2-kb regions upstream of the TSS), ranked and binned by the magnitude of change in H3K27ac signal intensity upon EWS-FLI1 knockdown.

(C) Line chart showing the proportion of differential H3K27ac peaks that overlap with EWS-FLI1 binding sites, ranked by magnitude of the change in H3K27ac signal intensity upon EWS-FLI1 knockdown. The percentages (y axis) refer to the top-N peaks up to the indicated rank (x axis).

(D) Line chart illustrating the strong enrichment of H3K27ac intensity at 697 super-enhancers (highlighted in red). Super-enhancers were annotated with ROSE (Lovén et al., 2013; Whyte et al., 2013).

(E) Genome browser screenshots showing examples of a typical enhancer (top) and a super-enhancer (bottom). These enhancer and super-enhancer elements (gray bars) are cell-type specific when compared to multi-tissue H3K27ac data from the ENCODE project (bottom track).

*(legend continued on next page)*

that overlapped with EWS-FLI1 binding sites (Figure S5F). Interestingly, EWS-FLI1 binding sites and EWS-FLI1-correlated H3K27ac peaks were both more likely than other H3K27ac peaks to be located in super-enhancers (Figure S5G); in addition, more than half of the super-enhancers in EWS-FLI1-expressing cells contained at least one EWS-FLI1 binding event, and a similar fraction contained at least one EWS-FLI1-correlated H3K27ac peak (Figure S5H).

To assess the cell-type specificity of EWS-FLI1-correlated H3K27ac peaks, we studied their association with active regulatory elements in 72 human cell types, based on DNase hypersensitivity data (Sheffield et al., 2013). Our analysis uncovered a striking trend toward high cell-type specificity among the strongest EWS-FLI1-correlated, EWS-FLI1-bound, and super-enhancer-associated H3K27ac peaks (Figure 6F). This trend was already visible for H3K27ac peaks in promoters and in typical enhancers, but the association with super-enhancers and overlap with EWS-FLI1 binding sites further increased the cell-type specificity (Figure 6G). Most notably, 37% of the 2,322 strongest EWS-FLI1-correlated H3K27ac enhancer peaks overlapped with DNase-hypersensitive sites present only in the one EWS-FLI1 expressing cell line that was part of the DNase dataset (SK-N-MC) and were thus unique to Ewing sarcoma. In summary, these results provide strong evidence that EWS-FLI1 not only regulates genes and promoters but also establishes highly cell-type-specific enhancers and super-enhancers in EWS-FLI1-expressing cells.

## DISCUSSION

### Global Epigenome Dynamics in EWS-FLI1-Dependent Cells

In this study, we used a Ewing sarcoma cell line with tunable EWS-FLI1 expression to uncover connections between this oncogenic fusion protein and the epigenome. Based on both ChIP-seq and western blots, H3K27ac emerged as the mark that was most strongly affected by EWS-FLI1 knockdown. 33,170 H3K27ac peaks were significantly altered throughout the genome (Figure 7A), and 80% of the most strongly EWS-FLI1-correlated H3K27ac peaks were bound by EWS-FLI1 (Figure 6C). In contrast, H3K27ac levels in regions that were not bound by EWS-FLI1 were either correlated or anticorrelated with EWS-FLI1 expression and likely driven by indirect mechanisms of transcription regulation. Similar but weaker patterns of EWS-FLI1 association were also observed for H3K4me3 and H3K4me1 (Figure 7A), while the other studied histone marks did not show widespread changes upon EWS-FLI1 knockdown. Differences in response to EWS-FLI1 knockdown were also notably absent for DNA methylation (Figure 7B). Although this mark was anticorrelated with EWS-FLI1 binding (Figure 2E), no changes in DNA methylation patterns were observed 53 hr after knockdown—at a time point that should be sufficient for de novo methylation to have at least started. Hence, it seems possible that Ewing sarcoma cells retain an epigenetic memory of prior EWS-FLI1 binding as part of their DNA methylation patterns.

### Promoter Regulation by EWS-FLI1

Chromatin-based promoter analysis identified four clusters of EWS-FLI1-correlated transcripts and four clusters of EWS-FLI1-anticorrelated transcripts. These clusters define three different regulatory modes (Figure 4). First, EWS-FLI1-correlated cluster 1 genes, such as the cell-cycle regulator and proto-oncogene *CCND1* (Sanchez et al., 2008), were expressed even in the EWS-FLI1-low state and carried all histone marks of active promoters, but their H3K27ac and transcription levels were higher in the EWS-FLI1-high state (Figure 7C). Many of the correlated cluster 1 genes were associated with cell proliferation, suggesting that increased H3K27ac at widely expressed genes may drive the rapid proliferation of EWS-FLI1-expressing cells. Second, the genes in EWS-FLI1-correlated clusters 2 and 3 (Figure 4A) were more tissue specific than genes in correlated cluster 1, and they lacked active promoter marks such as H3K27ac and/or H3K4me3 in the EWS-FLI1-low state. EWS-FLI1-dependent establishment of the missing histone marks may aberrantly activate these genes, as illustrated by the hematopoietic proto-oncogene *MYB* that was specifically expressed in the EWS-FLI1-high state (Figure 7D). Third, EWS-FLI1-anticorrelated genes had higher H3K27ac levels in the EWS-FLI1-low state independent of their cluster association and H3K27ac level in the EWS-FLI-high state (Figure 4D), which is illustrated by the AP-1 component FOSL1 (Figure 7E). Intriguingly, we observed broad enrichment for AP-1 binding in other cell types among EWS-FLI1-anticorrelated gene promoters (Figure 4B), suggesting that EWS-FLI1 may repress a sizable fraction of its target genes by interfering with the activating role of AP-1. HDACs seem to play a role in the process, given the upregulation of EWS-FLI1-anticorrelated genes (clusters 2, 3, and 4) upon treatment with HDAC inhibitors specifically in the EWS-FLI1-high state (Figure 5D).

### Enhancer and Super-Enhancer Regulation by EWS-FLI1

Our analysis of H3K27ac resulted in a genome-wide catalog of EWS-FLI1-associated enhancers and super-enhancers. Strikingly, the most dynamic enhancers upon EWS-FLI1 knockdown were also the most cell-type specific (Figure 6), which is illustrated by the absence of any ENCODE signal for H3K27ac at the EWS-FLI1-bound enhancers upstream of the *CCND1* promoter (Figure 7C). A large percentage of the observed EWS-FLI1-correlated H3K27ac peaks overlapped exclusively with regulatory elements in SK-N-MC cells, which is the one

(F) Bar plots showing the degree to which differential H3K27ac peaks co-localize with open chromatin regions in other cell types. An ENCODE dataset with DNase-hypersensitivity data for 72 cell types was used to quantify cell type specificity (y axis). High values indicate regions with widespread or ubiquitously open chromatin, whereas low values indicate enhancers that are highly tissue specific. The differential H3K27ac peaks are ranked and binned according to the magnitude of change in signal intensity upon EWS-FLI1 knockdown (x axis).

(G) Q-Q plots showing the difference in tissue specificity for all EWS-FLI1-correlated enhancers versus those in super-enhancers (top) and those overlapping EWS-FLI1 peaks (bottom).

See also Figure S5.

OPEN ACCESS
CellPress

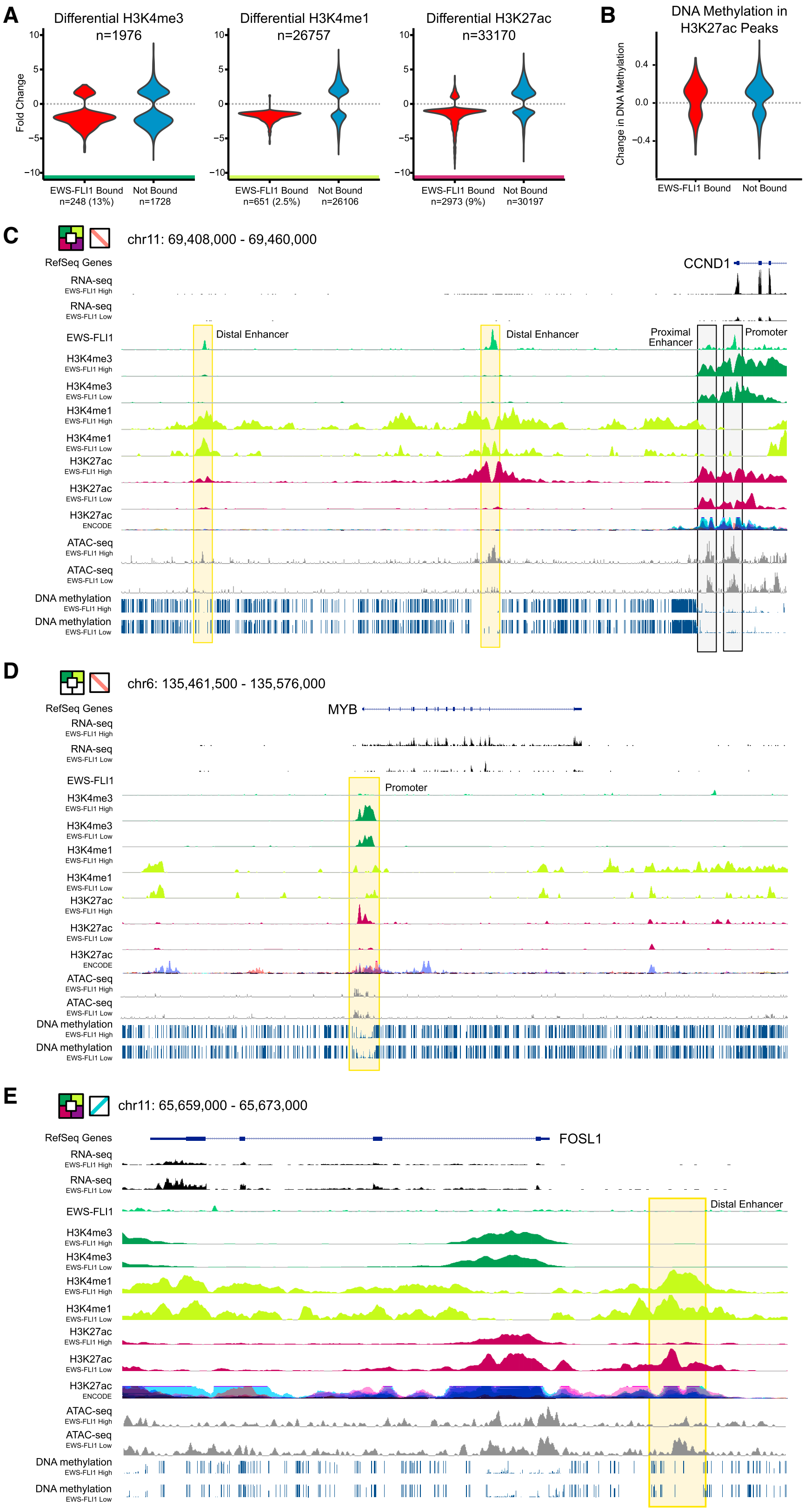


**Figure 7. Epigenome Dynamics and Modes of Transcriptional Regulation in EWS-FLI1-Dependent Cells**

(A) Violin plots showing the distribution of fold change upon EWS-FLI1 knockdown for the three most strongly EWS-FLI1-correlated histone marks (H3K4me3, H3K4me1, and H3K27ac). Data are shown separately for peaks that are bound or not bound by EWS-FLI1, and the difference between the two distributions is highly significant for all three histone marks ($p < 10^{-16}$, Kolmogorov-Smirnov test).

(B) Violin plots showing the distribution of differential DNA methylation at H3K27ac peaks that are bound or not bound by EWS-FLI1. The difference between the two distributions is not significant ($p > 0.05$, Kolmogorov-Smirnov test).

(C) Genome browser screenshot showing *CCND1*, an EWS-FLI1-correlated cluster 1 gene with an established role in cell proliferation. Yellow boxes indicate EWS-FLI1-correlated H3K27ac peaks at EWS-FLI1-bound enhancers, which are highly cell-type specific relative to the ENCODE H3K27ac track. Black boxes highlight H3K27ac peaks associated with EWS-FLI1 binding in the promoter region, which are not cell-type specific.

(D) Genome browser screenshot showing *MYB*, an EWS-FLI1-correlated cluster 2 gene that is a known proto-oncogene of the hematopoietic lineage. The H3K27ac peak in the promoter region (yellow box) is EWS-FLI1 correlated and lost after knockdown, but it is not directly bound by EWS-FLI1.

(E) Genome browser screenshot showing *FOSL1*, an EWS-FLI1-anticorrelated cluster 1 gene that encodes a component of the AP-1 transcription factor. The yellow box indicates EWS-FLI1-anti-correlated H3K27ac peaks at a constitutive enhancer element, while the promoter has active histone marks even in the EWS-FLI1-high state.

EWS-FLI1-expressing cell line in a published catalog with DNase hypersensitivity data for 72 human cell types ($p < 10^{-100}$, Fisher's exact test). EWS-FLI1 also appears to contribute to cell-type-specific enhancer signatures by converting broadly active enhancers to a poised state, as illustrated by the enhancer element upstream of the *FOSL1* locus (Figure 7E). While our study was in revision, Riggi et al. published conclusive evidence that EWS-FLI1 binds *cis*-regulating elements shared by Ewing sarcoma cell lines and primary tumors (Riggi et al., 2014), suggesting that our observations are not only limited to cell lines but also relevant for Ewing sarcoma biology in patients.

In summary, the establishment and analysis of reference epigenomes for EWS-FLI1-high and EWS-FLI1-low cells not only constitutes a first comprehensive epigenome dataset for a solid cancer that is driven by an oncogenic fusion protein, but it also provides relevant insights into the mechanisms by which a single oncogenic event has shaped a conductive epigenome. The dataset is available for interactive exploration and data download (http://tomazou2015.computational-epigenetics.org), thus providing a genome-wide reference that can guide future work on regulatory mechanisms and epigenetic drug effects in Ewing sarcoma.

## EXPERIMENTAL PROCEDURES

For full details, see the Supplemental Experimental Procedures.

### Epigenome Mapping

A673 cells were cultured as previously described (Carrillo et al., 2007). DNA, chromatin, RNA, and protein were collected following standard procedures from untreated cells (EWS-FLI1-high state) and 53 hr after adding doxycycline to the media (EWS-FLI1-low state). To assess DNA methylation, WGBS and RRBS experiments were performed using custom protocols (see the Supplemental Experimental Procedures for details), and the data of both assays were merged. ChIP-seq experiments were performed using the iDeal ChIP-seq kit (Diagenode) according to manufacturer instructions. ATAC-seq was performed as previously described (Buenrostro et al., 2013) with minor adaptations for A673 cells. RNA-seq used Illumina kits for strand-specific library preparation. All sequencing was performed by the Biomedical Sequencing Facility at CeMM using Illumina HiSeq 2000 sequencers.

The epigenome maps as well as the raw and processed data underlying the presented analyses are available online at http://tomazou2015.computational-epigenetics.org.

### Epigenetic Gene Clustering

We clustered all EWS-FLI1-associated genes based on the ChIP-seq signal intensity of five promoter-associated histone marks in the vicinity of the TSS (excluding H3K36me3 and H3K9me3, which were rarely present in promoter regions), using a two-step semi-supervised clustering method. Multinomial logistic regression models were trained based on expert-curated examples and used to classify EWS-FLI1-regulated genes derived from the RNA-seq data. We trained separate classifiers for identifying clusters among the EWS-FLI1-correlated genes and among the EWS-FLI1-anticorrelated genes.

### Enrichment Analysis

To functionally annotate the gene clusters, we identified significant overlap between gene sets derived from our dataset and gene sets obtained from several public databases (see the Supplemental Experimental Procedures for details). In addition to gene-based enrichments, we also developed a method for region-based enrichment analysis called location overlap analysis (LOLA; http://lola.computational-epigenetics.org), and we identified sets of functional elements that significantly co-localized with the promoters of a given gene cluster. We used Fisher's exact test to establish significance, and odds ratios as well as p values were used to rank the results. Comparisons were done (1) against the background of all genes identified by our RNA-seq analysis to identify enrichment patterns among EWS-FLI1-regulated genes, (2) against the background of genes in other clusters to identify cluster-specific enrichment patterns separately for EWS-FLI1-correlated and EWS-FLI1-anticorrelated genes, and (3) against the background of genes in the same cluster but with opposite direction of EWS-FLI1 regulation (correlated or anticorrelated) to identify upregulation- versus downregulation-specific enrichment patterns separately for each cluster.

## SUPPLEMENTAL INFORMATION

Supplemental Information includes Supplemental Experimental Procedures, five figures, and one table and can be found with this article online at http://dx.doi.org/10.1016/j.celrep.2015.01.042.

## AUTHOR CONTRIBUTIONS

E.M.T., N.C.S., C.B., and H.K. designed the study. E.M.T. performed the experiments and contributed to the data analysis. N.C.S. performed the data analysis. C.S. and P.D. contributed to the experiments. M.S. and A.S. contributed to the data analysis. S.K. provided relevant materials and drug-related expertise. E.M.T., N.C.S., C.B., and H.K. wrote the manuscript.

## ACKNOWLEDGMENTS

This study was supported by a next-generation sequencing grant to E.M.T., funded through a charitable donation of Kapsch group (http://www.kapsch.net/kapschgroup), and by a Lise Meitner Fellowship of the Austrian Science Fund (FWF, M1448-B13) to E.M.T. N.C.S. was supported by long-term fellowships of the European Molecular Biology Organization (ALTF 1431-2013) and the Human Frontier Science Program (LT000211/2014). C.S. was supported by a Feodor Lynen Fellowship of the Alexander von Humboldt Foundation. The work was performed in the context of the European Union's FP7 projects ASSET (grant agreement no. 259348) and BLUEPRINT (grant agreement no. 282510). We would like to thank the team of the Biomedical Sequencing Facility at CeMM for their support with next-generation sequencing and the members of the Bock and Kovar labs for feedback on the manuscript.

Received: August 2, 2014
Revised: November 17, 2014
Accepted: January 15, 2015
Published: February 27, 2015

## REFERENCES

Arnaldez, F.I., and Helman, L.J. (2014). New strategies in ewing sarcoma: lost in translation? Clin. Cancer Res. *20*, 3050–3056.

Bailly, R.A., Bosselut, R., Zucman, J., Cormier, F., Delattre, O., Roussel, M., Thomas, G., and Ghysdael, J. (1994). DNA-binding and transcriptional activation properties of the EWS-FLI-1 fusion protein resulting from the t(11;22) translocation in Ewing sarcoma. Mol. Cell. Biol. *14*, 3230–3241.

Bernstein, M., Kovar, H., Paulussen, M., Randall, R.L., Schuck, A., Teot, L.A., and Juergens, H. (2006). Ewing's sarcoma family of tumors: current management. Oncologist *11*, 503–519.

Bernstein, B.E., Birney, E., Dunham, I., Green, E.D., Gunter, C., and Snyder, M.; ENCODE Project Consortium (2012). An integrated encyclopedia of DNA elements in the human genome. Nature *489*, 57–74.

Bilke, S., Schwentner, R., Yang, F., Kauer, M., Jug, G., Walker, R.L., Davis, S., Zhu, Y.J., Pineda, M., Meltzer, P.S., and Kovar, H. (2013). Oncogenic ETS fusions deregulate E2F3 target genes in Ewing sarcoma and prostate cancer. Genome Res. *23*, 1797–1809.

Brohl, A.S., Solomon, D.A., Chang, W., Wang, J., Song, Y., Sindiri, S., Patidar, R., Hurd, L., Chen, L., Shern, J.F., et al. (2014). The genomic landscape of the Ewing Sarcoma family of tumors reveals recurrent STAG2 mutation. PLoS Genet. *10*, e1004475.

Buenrostro, J.D., Giresi, P.G., Zaba, L.C., Chang, H.Y., and Greenleaf, W.J. (2013). Transposition of native chromatin for fast and sensitive epigenomic profiling of open chromatin, DNA-binding proteins and nucleosome position. Nat. Methods *10*, 1213–1218.

Carrillo, J., García-Aragoncillo, E., Azorín, D., Agra, N., Sastre, A., González-Mediero, I., García-Miguel, P., Pestaña, A., Gallego, S., Segura, D., and Alonso, J. (2007). Cholecystokinin down-regulation by RNA interference impairs Ewing tumor growth. Clin. Cancer Res. *13*, 2429–2440.

Chen, J., Odenike, O., and Rowley, J.D. (2010). Leukaemogenesis: more than mutant genes. Nat. Rev. Cancer *10*, 23–36.

Creyghton, M.P., Cheng, A.W., Welstead, G.G., Kooistra, T., Carey, B.W., Steine, E.J., Hanna, J., Lodato, M.A., Frampton, G.M., Sharp, P.A., et al. (2010). Histone H3K27ac separates active from poised enhancers and predicts developmental state. Proc. Natl. Acad. Sci. USA *107*, 21931–21936.

Crompton, B.D., Stewart, C., Taylor-Weiner, A., Alexe, G., Kurek, K.C., Calicchio, M.L., Kiezun, A., Carter, S.L., Shukla, S.A., Mehta, S.S., et al. (2014). The genomic landscape of pediatric Ewing sarcoma. Cancer Discov. *4*, 1326–1341.

Delattre, O., Zucman, J., Melot, T., Garau, X.S., Zucker, J.M., Lenoir, G.M., Ambros, P.F., Sheer, D., Turc-Carel, C., Triche, T.J., et al. (1994). The Ewing family of tumors—a subgroup of small-round-cell tumors defined by specific chimeric transcripts. N. Engl. J. Med. *331*, 294–299.

ENCODE Project Consortium (2012). An integrated encyclopedia of DNA elements in the human genome. Nature *489*, 57–74.

Hahm, K.B., Cho, K., Lee, C., Im, Y.H., Chang, J., Choi, S.G., Sorensen, P.H., Thiele, C.J., and Kim, S.J. (1999). Repression of the gene encoding the TGF-beta type II receptor is a major target of the EWS-FLI1 oncoprotein. Nat. Genet. *23*, 222–227.

Hnisz, D., Abraham, B.J., Lee, T.I., Lau, A., Saint-André, V., Sigova, A.A., Hoke, H.A., and Young, R.A. (2013). Super-enhancers in the control of cell identity and disease. Cell *155*, 934–947.

Huether, R., Dong, L., Chen, X., Wu, G., Parker, M., Wei, L., Ma, J., Edmonson, M.N., Hedlund, E.K., Rusch, M.C., et al. (2014). The landscape of somatic mutations in epigenetic regulators across 1,000 paediatric cancer genomes. Nat. Commun. *5*, 3630.

Kauer, M., Ban, J., Kofler, R., Walker, B., Davis, S., Meltzer, P., and Kovar, H. (2009). A molecular function map of Ewing's sarcoma. PLoS ONE *4*, e5415.

Kovar, H. (2010). Downstream EWS/FLI1 - upstream Ewing's sarcoma. Genome Med. *2*, 8.

Lawrence, M.S., Stojanov, P., Polak, P., Kryukov, G.V., Cibulskis, K., Sivachenko, A., Carter, S.L., Stewart, C., Mermel, C.H., Roberts, S.A., et al. (2013). Mutational heterogeneity in cancer and the search for new cancer-associated genes. Nature *499*, 214–218.

Lovén, J., Hoke, H.A., Lin, C.Y., Lau, A., Orlando, D.A., Vakoc, C.R., Bradner, J.E., Lee, T.I., and Young, R.A. (2013). Selective inhibition of tumor oncogenes by disruption of super-enhancers. Cell *153*, 320–334.

Macconaill, L.E., and Garraway, L.A. (2010). Clinical implications of the cancer genome. J. Clin. Oncol. *28*, 5219–5228.

Mitelman, F., Johansson, B., and Mertens, F. (2004). Fusion genes and rearranged genes as a linear function of chromosome aberrations in cancer. Nat. Genet. *36*, 331–334.

Ohno, T., Rao, V.N., and Reddy, E.S. (1993). EWS/Fli-1 chimeric protein is a transcriptional activator. Cancer Res. *53*, 5859–5863.

Prieur, A., Tirode, F., Cohen, P., and Delattre, O. (2004). EWS/FLI-1 silencing and gene profiling of Ewing cells reveal downstream oncogenic pathways and a crucial role for repression of insulin-like growth factor binding protein 3. Mol. Cell. Biol. *24*, 7275–7283.

Rada-Iglesias, A., Bajpai, R., Swigut, T., Brugmann, S.A., Flynn, R.A., and Wysocka, J. (2011). A unique chromatin signature uncovers early developmental enhancers in humans. Nature *470*, 279–283.

Riggi, N., Suvà, M.L., Suvà, D., Cironi, L., Provero, P., Tercier, S., Joseph, J.M., Stehle, J.C., Baumer, K., Kindler, V., and Stamenkovic, I. (2008). EWS-FLI-1 expression triggers a Ewing's sarcoma initiation program in primary human mesenchymal stem cells. Cancer Res. *68*, 2176–2185.

Riggi, N., Knoechel, B., Gillespie, S.M., Rheinbay, E., Boulay, G., Suvà, M.L., Rossetti, N.E., Boonseng, W.E., Oksuz, O., Cook, E.B., et al. (2014). EWS-FLI1 utilizes divergent chromatin remodeling mechanisms to directly activate or repress enhancer elements in Ewing sarcoma. Cancer Cell *26*, 668–681.

Rivera, C.M., and Ren, B. (2013). Mapping human epigenomes. Cell *155*, 39–55.

Sanchez, G., Bittencourt, D., Laud, K., Barbier, J., Delattre, O., Auboeuf, D., and Dutertre, M. (2008). Alteration of cyclin D1 transcript elongation by a mutated transcription factor up-regulates the oncogenic D1b splice isoform in cancer. Proc. Natl. Acad. Sci. USA *105*, 6004–6009.

Sankar, S., Bell, R., Stephens, B., Zhuo, R., Sharma, S., Bearss, D.J., and Lessnick, S.L. (2013). Mechanism and relevance of EWS/FLI-mediated transcriptional repression in Ewing sarcoma. Oncogene *32*, 5089–5100.

Sheffield, N.C., Thurman, R.E., Song, L., Safi, A., Stamatoyannopoulos, J.A., Lenhard, B., Crawford, G.E., and Furey, T.S. (2013). Patterns of regulatory activity across diverse human cell types predict tissue identity, transcription factor binding, and long-range interactions. Genome Res. *23*, 777–788.

Smith, R., Owen, L.A., Trem, D.J., Wong, J.S., Whangbo, J.S., Golub, T.R., and Lessnick, S.L. (2006). Expression profiling of EWS/FLI identifies NKX2.2 as a critical target gene in Ewing's sarcoma. Cancer Cell *9*, 405–416.

Stadler, M.B., Murr, R., Burger, L., Ivanek, R., Lienert, F., Schöler, A., van Nimwegen, E., Wirbelauer, C., Oakeley, E.J., Gaidatzis, D., et al. (2011). DNA-binding factors shape the mouse methylome at distal regulatory regions. Nature *480*, 490–495.

Tirode, F., Laud-Duval, K., Prieur, A., Delorme, B., Charbord, P., and Delattre, O. (2007). Mesenchymal stem cell features of Ewing tumors. Cancer Cell *11*, 421–429.

Tirode, F., Surdez, D., Ma, X., Parker, M., Le Deley, M.C., Bahrami, A., Zhang, Z., Lapouble, E., Grossetête-Lalami, S., Rusch, M., et al.; St. Jude Children's Research Hospital–Washington University Pediatric Cancer Genome Project and the International Cancer Genome Consortium (2014). Genomic landscape of Ewing sarcoma defines an aggressive subtype with co-association of STAG2 and TP53 mutations. Cancer Discov. *4*, 1342–1353.

Whyte, W.A., Orlando, D.A., Hnisz, D., Abraham, B.J., Lin, C.Y., Kagey, M.H., Rahl, P.B., Lee, T.I., and Young, R.A. (2013). Master transcription factors and mediator establish super-enhancers at key cell identity genes. Cell *153*, 307–319.

Cell Reports
Supplemental Information

# Epigenome Mapping Reveals Distinct Modes of Gene Regulation and Widespread Enhancer Reprogramming by the Oncogenic Fusion Protein EWS-FLI1

Eleni M. Tomazou, Nathan C. Sheffield, Christian Schmidl, Michael Schuster, Andreas Schönegger, Paul Datlinger, Stefan Kubicek, Christoph Bock, and Heinrich Kovar

**SUPPLEMENTAL INFORMATION**

## Supplemental Table Legends

Table S1. Epigenome sequencing statistics, related to Figure 1 (provided as an Excel file)

## Supplemental Figures

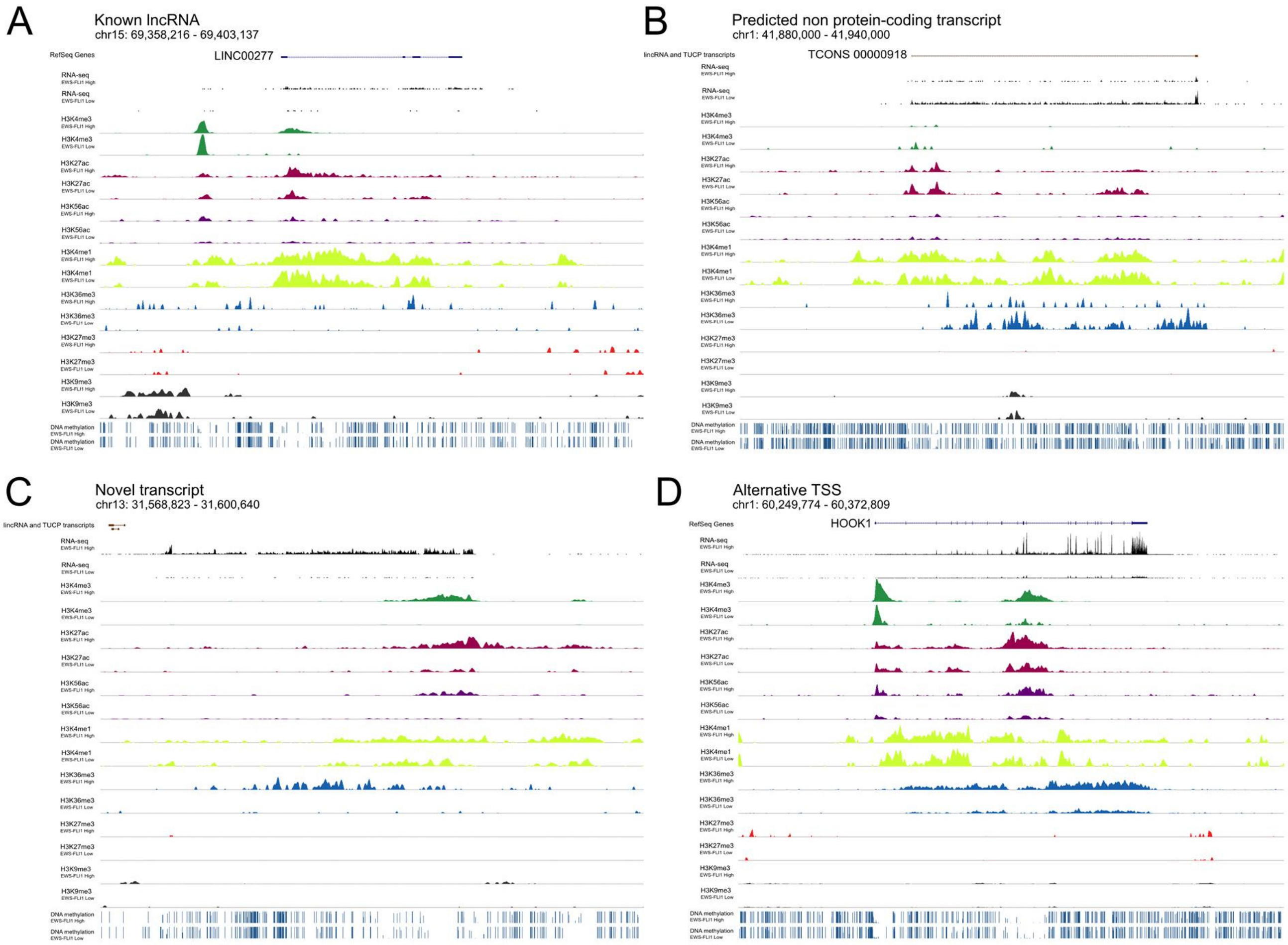


**Figure S1: Epigenome maps for EWS-FLI1 regulated transcripts, Related to Figure 1**

Genome browser screenshots of epigenome maps for the A673 cell line, showing the genomic loci of four EWS-FLI1 regulated transcripts: a known lincRNA (A); a predicted non-coding transcript (B); a novel transcript (C); and an alternative transcription start site of a known gene (D). All transcripts are differentially expressed between the EWS-FLI1 high state (before EWS-FLI1 knockdown) and the EWS-FLI1 low state (after EWS-FLI1 knockdown). This figure illustrates the use of chromatin maps for discovering EWS-FLI1 regulated transcripts. All data are available online at http://tomazou2015.computational-epigenetics.org.

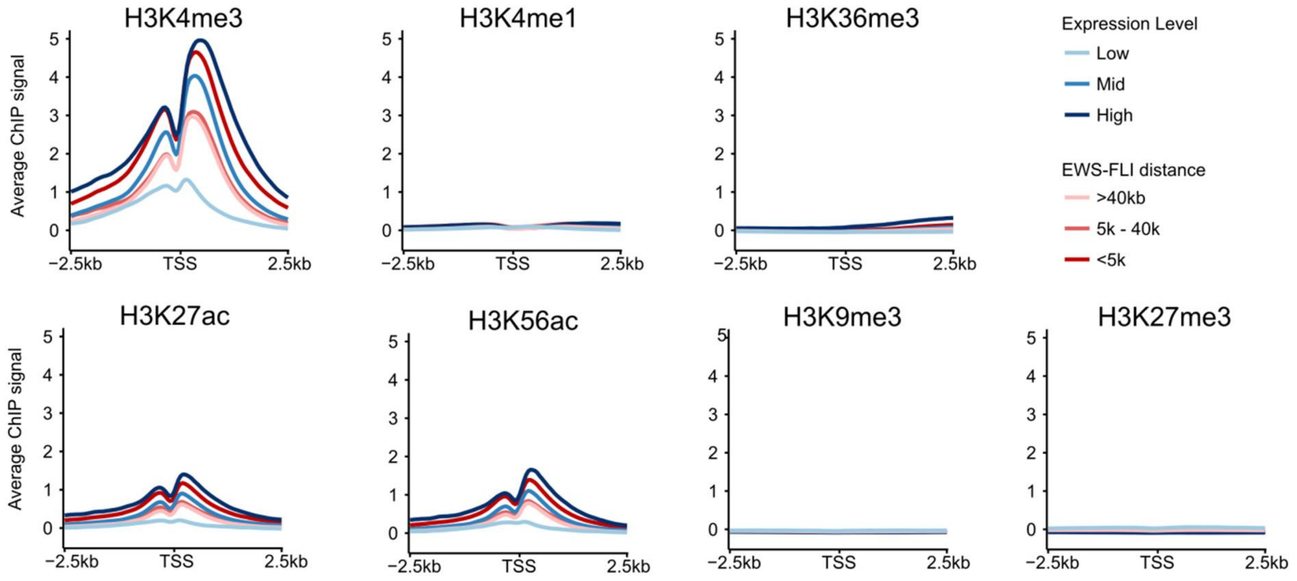


**Figure S2: Distribution of histone modifications around transcription start sites, Related to Figure 2**

Composite plots showing the aggregate ChIP-seq enrichment for each histone modification around the TSS, stratified in two ways: by the expression levels of associated genes (shades of blue); and by the distance to the nearest EWS-FLI1 binding peak (shades of red). These plots are the same as those shown in Figure 2A of the main manuscript, except that here the y-axis is scaled in the same way across all plots (which makes it easier to see the strong differences in observed ChIP-seq signal between different histone marks).

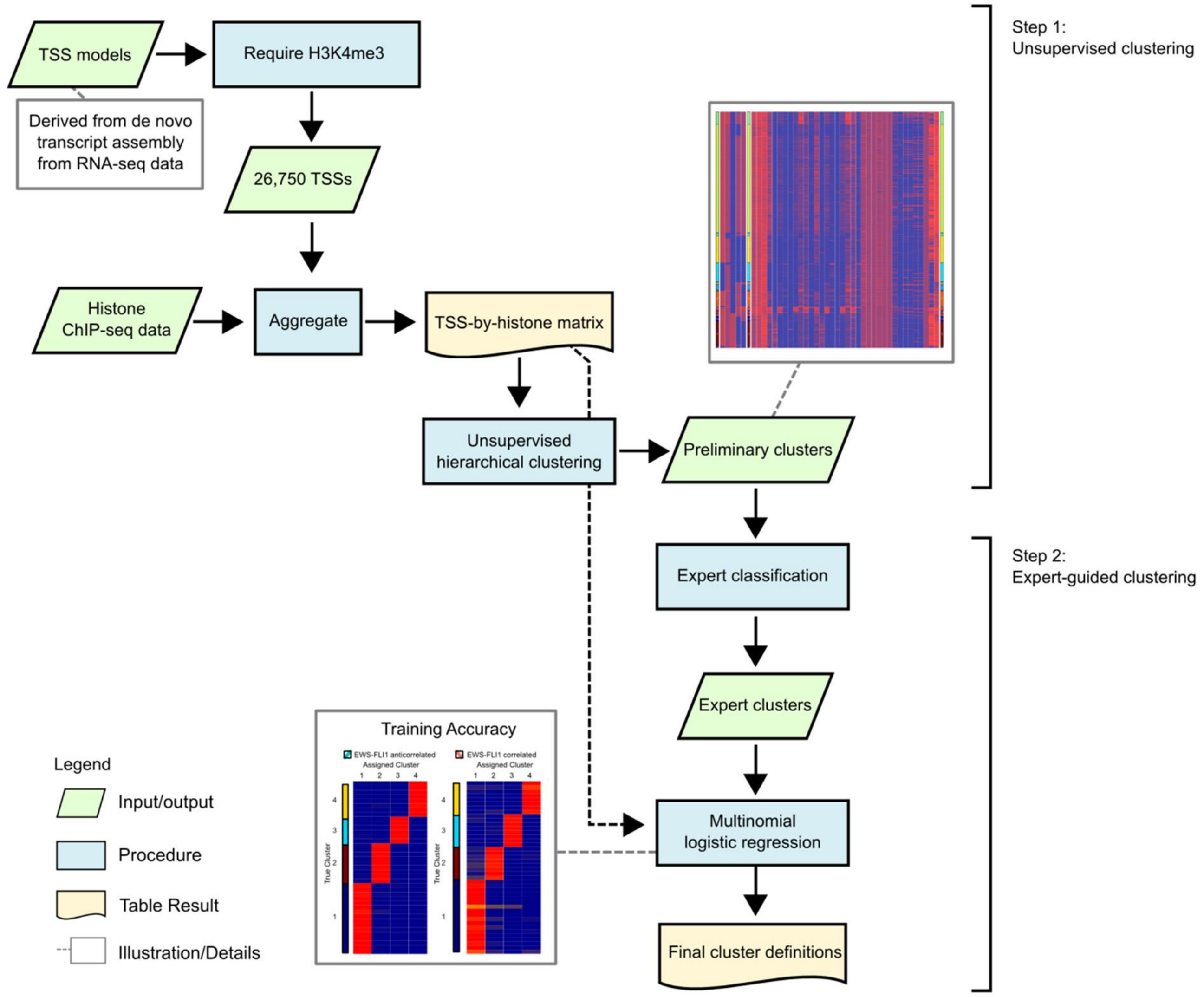


**Figure S3: Workflow for chromatin-based clustering of EWS-FLI1 regulated transcripts, Related to Figure 3**

Schematic diagram illustrating the semi-supervised clustering method that was used to group EWS-FLI1 regulated genes into clusters based on their histone marks. After annotating each TSS with the quantitative enrichment for promoter-associated histone marks, the TSSs were clustered by unsupervised hierarchical clustering. The resulting preliminary clusters were examined to define a set of expert examples, which provided the basis for training a multinomial logistic regression model. Using the model to predict cluster assignments on the original dataset resulted in the final cluster definitions. More details of the algorithm are given in the Supplemental Experimental Procedures.

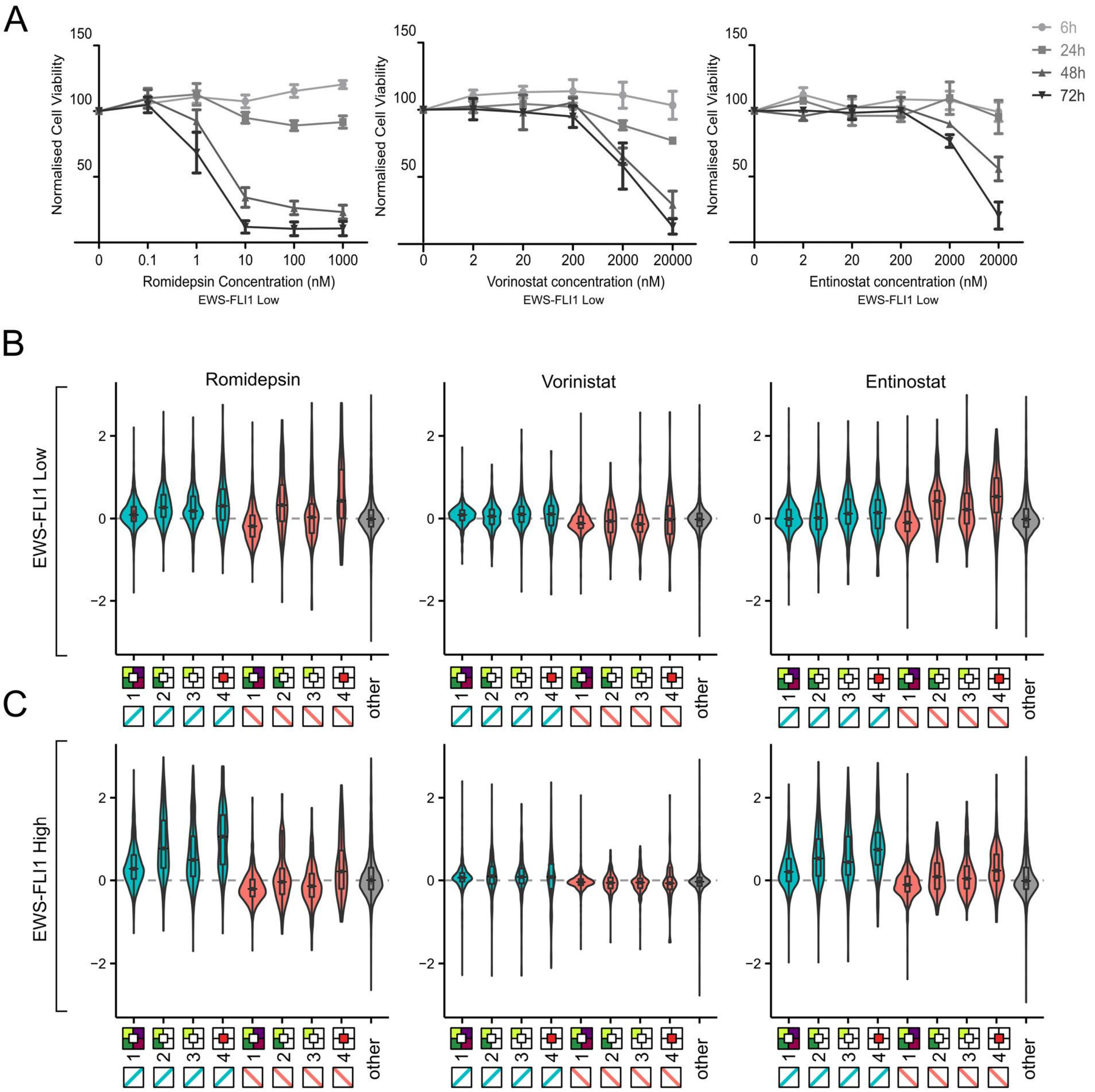


**Figure S4: Response to treatment with HDAC inhibitors, Related to Figure 5**

A: Dose-response curves showing the cell viability effect of three histone deacetylase inhibitors (HDACi) at different concentrations (x-axis) and time points (greyscale curves). Data are shown for the EWS-FLI1 low state. Cell viability was measured by the MTT assay and error bars represent the SEM of triplicate experiments.

B: Violin plots visualizing gene expression changes in response to HDAC inhibitor treatment in the EWS-FLI1 low state. The y-axis shows fold change, and the x-axis divides the genes by cluster and direction of response to EWS-FLI1 knockdown, as indicated by the annotation icons. Gray bars represent all other genes (those not responding to EWS-FLI1 knockdown).

C: Same as panel B, but for the EWS-FLI1 high state.

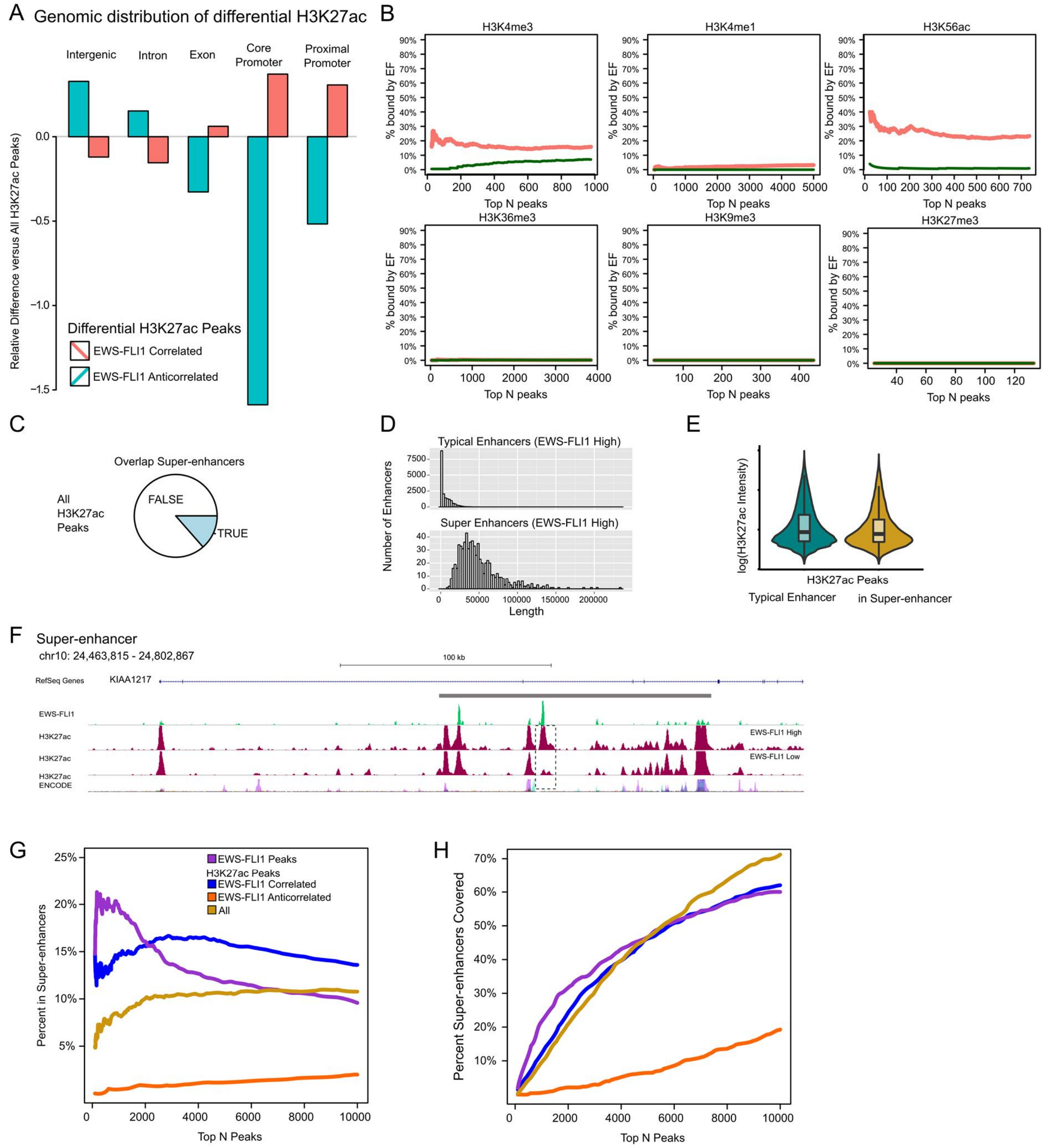


**Figure S5: Enhancers & super-enhancers in EWS-dependent cells, Related to Figure 6**

A: Bar charts showing the localization of differential H3K27ac peaks compared to non-differential H3K27ac peaks. The y-axis shows log fold changes indicative of relative enrichment or depletion.

B: Line charts analogous to Figure 6C, showing the histone marks other than H3K27ac. For each histone mark, differential peaks are called and then ranked by magnitude of change. These peaks are then tested for overlap with EWS-FLI1 binding site, and a curve showing the percentage of differential histone peaks covering an EWS-FLI1 binding site for the top N peaks is shown. Red line: EWS-FLI1 correlated; green line: EWS-FLI1 anticorrelated.

C: Pie chart illustrating the percentage of H3K27ac peaks that overlap with super-enhancers.

D: Histograms of enhancer and super-enhancer length distributions, showing that super-enhancers are longer than typical enhancers.

E: Violin plots showing that H3K27ac peaks in super-enhancers are in general not individually stronger than H3K27ac peaks located outside of super-enhancers.

F: Genome browser screenshot showing a super-enhancer (grey box) with a localized change in H3K27ac overlapping an EWS-FLI1 binding peak (dotted box). This peak is cell-type specific when compared with cross-tissue H3K27ac data from the ENCODE project (bottom track).

G: Line graph showing the percentage of peaks in super-enhancers for four sets of regions: All H3K27ac peaks (yellow), EWS-FLI1 correlated differential H3K27ac peaks (blue), EWS-FLI1 anticorrelated differential H3K27ac peaks (red), and EWS-FLI1 peaks (purple). Each set of regions is ranked by signal intensity (decreasing from left to right), and the y-axis shows the cumulative percentage of Top N peaks that overlap with a super-enhancer.

H: Line graph showing the percentage of super-enhancers that contain at least one of the top N peaks for each of the four sets of regions described for panel F.

## Supplemental Website

The epigenome maps as well as the raw and processed data underlying the presented analyses are available online: http://tomazou2015.computational-epigenetics.org.

## Supplemental Experimental Procedures

### *Cell Culture*

We used a subclone of the Ewing sarcoma cell line A673 that has been stably transfected with a doxycycline-inducible shRNA against the EWS-FLI1 fusion protein (Carrillo et al., 2007). Cells were propagated in DMEM+GlutaMax supplemented with 10% fetal bovine serum, 50μg/ml Zeocin (InvivoGen, ant-zn-5p) and 2μg/ml Blasticidin (InvivoGen, ant-bl-10p). For the EWS-FLI1 shRNA induction, 1μg/ml doxycycline (Sigma, D9891) was added to the media for 53 hours. For the additional Ewing sarcoma cell lines TC252, SK-N-MC and STA-ET-7.2, the EWS-FLI1 knockdown was performed using transient transfection with shRNA constructs as previously described (Ban et al., 2008).

### *Bisulfite sequencing experiments*

For reduced representation bisulfite sequencing (RRBS), 100 ng of genomic DNA were digested for 12 hours at 37°C with 20 units of MspI (New England Biolabs, R0106L) in 30 μl of 1x NEB buffer 2. Fill-in and A-tailing were performed by addition of Klenow Fragment 3’ > 5’ exo- (New England Biolabs, M0212L) and dNTP mix (10 mM dATP, 1 mM dCTP, 1 mM dGTP). After ligation to methylated Illumina TruSeq LT v2 adaptors using Quick Ligase (New England Biolabs, M2200L), the libraries were size selected by performing a 0.75x clean-up with AMPure XP beads (Beckman Coulter, A63881). The four libraries were pooled in equal amounts based on qPCR data and bisulfite converted using the EZ DNA Methylation Direct

Kit (Zymo Research, D5020) with changes to the manufacturer's protocol: conversion reagent was used at 0.9x concentration, incubation performed for 20 cycles of 1 min at 95°C, 10 min at 60°C and desulphonation time extended to 30 min. Bisulfite-converted libraries were enriched for 17 cycles using PfuTurbo Cx Hotstart DNA Polymerase (Agilent, 600412). After a 2x AMPure XP clean-up, quality control was performed by a Qubit dsDNA HS (Life Technologies, Q32854) and Experion DNA 1k assay (BioRad, 700-7107).

For whole genome bisulfite sequencing (WGBS), 3 µg of DNA were fragmented by Covaris S2 system two times for 60 seconds at duty cycle 20%, intensity 4, and 200 cycles per burst. Fill-in, A-tailing and adapter ligation were performed by using the TruSeq DNA sample preparation kit (Illumina) following the manufacturer's protocol. After each step fragments were purified by performing a 0.8x clean-up with AMPure XP beads (Beckman Coulter, A63881). Bisulfite conversion was performed by using the EpiTect Bisulfite kit (Qiagen) following the protocol specified for DNA isolated from FFPE tissue samples. Quantitative real-time PCR (20 µl reaction) was performed to determine the minimal PCR cycle number for library amplification using 1 µl bisulfite-converted DNA. Bisulfite-converted libraries were enriched for the determined number of cycles using PfuTurbo Cx Hotstart DNA Polymerase (Agilent, 600412). After a 2x AMPure XP clean-up, quality control was performed by a Qubit dsDNA HS (Life Technologies, Q32854) and Experion DNA 1k assay (BioRad, 700-7107).

*ChIP-seq experiments*

Chromatin immunoprecipitation (ChIP) was performed using the iDeal ChIP-seq kit (Diagenode) following the manufacturer's instructions. One million cells were cross-linked with 1% formaldehyde for 8 min at room temperature with constant agitation followed by quenching with 125 mM glycine for 5 min at room temperature. Nuclei were isolated and chromatin was sheared using the truChIP Low Cell Chromatin Shearing Kit (Covaris) and an S220 Ultrasonicator from Covaris. DNA fragments were in the range of 200-700 base pairs. Chromatin was incubated with antibody overnight at 4°C, with constant agitation. The following antibodies were used: H3K4me3 (Diagenode, C15410003-50, pAb-003-050), H3K27me3 (Diagenode, C15410195, pAb-195-050), H3K4me1 (Diagenode, pAb-194-050), H3K27ac (Diagenode, pAb-196-050), H3K56ac (Active Motif, 39281), H3K9me3 (Diagenode, pAb-193-050), H3K36me3 (Diagenode, pAb-192-050). During ChIP-seq library preparation, DNA isolated from ChIP experiments as well as input control DNA were end repaired, A-tailed, ligated to barcoded Illumina adaptors, PCR-amplified, and pooled for sequencing.

*ATAC-seq*

Open chromatin mapping was performed with the assay for transposase accessible chromatin (ATAC-seq) as previously described (Buenrostro et al., 2013) with minor adaptations for A673 cells. In each experiment, $1x10^5$ cells were washed once in 50 µl PBS, resuspended in 50 µl ATAC-seq lysis buffer (10 mM Tris-HCl, pH 7.4, 10 mM NaCl, 3 mM $MgCl_2$ and 0.1% IGEPAL CA-630), and centrifuged for 10 min at 4 °C. Upon centrifugation, the pellet was washed briefly in 50 µl $MgCl_2$ buffer (10mM Tris pH8.0, 5mM $MgCl_2$) before incubating in the transposase reaction mix (25 µL 2× TD buffer, 2.5 µL transposase (Illumina) and 22.5 µL nuclease- free water) for 30 min at 37°C. After DNA purification with the MinElute kit (Qiagen), 1 µl of the eluted DNA was used in a qPCR reaction to estimate the optimum number of amplification cycles. Library amplification was followed by a SPRI size-selection to exclude

fragments larger than 1200bp. DNA concentration was measured with a Qubit fluorometer (Life Technologies).

*RNA-seq experiments*

Total RNA was isolated with TRIzol Reagent (Invitrogen). RNA amount was measured using Qubit 2.0 Fluorometric Quantitation system (Life Technologies), and RNA integrity number (RIN) was determined using Experion Automated Electrophoresis System (Bio-Rad). RNA-seq libraries for the reference transcriptomes of EWS-FLI1 high and EWS-FLI1 low cells were prepared using TruSeq Stranded Total RNA LT Ribo-Zero Human/Mouse/Rat sample preparation kit (Illumina). RNA-seq libraries for samples treated with HDAC inhibitors were prepared using a Sciclone NGS Workstation (PerkinElmer) and a Zepyhr NGS Workstation (PerkinElmer) with the TruSeq Stranded mRNA LT sample preparation kit (Illumina). Library amount and quality was determined using Qubit 2.0 Fluorometric Quantitation system (Life Technologies) and Experion Automated Electrophoresis System (Bio-Rad).

*Western blotting*

Western blots were carried out using standard protocols and with the same antibodies that were also used for the ChIP-seq experiments. In addition, antibodies for EWS-FLI1 (MyBio-Source, MBS300723) and beta Actin (Abcam, 8226) were used. Western blot signals were acquired with an Odyssey Li-COR laser scanning and imaging system (Li-COR).

*Drug treatment experiments*

Cell viability was measured by performing MTT colometric assay. A673 cells were resus-pended in fresh media containing one of the below drugs: Romidepsin (0.1 nM, 1 nM, 10 nM, 100 nM, and 1,000 nM), vorinostat (2 nM, 20 nM, 200 nM, 2,000 nM), entinostat (2 nM, 20 nM, 200 nM, 2,000 nM, and 20,000 nM) or vehicle (DMSO, 0.1%). Cells were plated in 96-well plates at 10,000 cells/well in a volume of 100 µl. Viability was measured after 6, 24, 48, and 72 hr incubations by addition of MTT solution (20 µl, 5 mg/ml) (Sigma, M2128) and luminescence measurement on an EnSpire multimode plate reader. For the EWS-FLI1 low state, inhibitors were added 24h after adding doxycycline to induce knockdown. All experiments were performed in three biological replicates (each biological replicate is the average of four technical replicates). Based on the dose-response curves, the following drug concentrations and time points were selected for measuring the transcriptome response by RNA-seq in two biological replicates: Romidepsin (1nM) for 24h, vorinostat (200 nM) for 24h, entinostat (2,000 nM) for 6h or vehicle (DMSO, 0.1%). Drugs were provided by the Platform Austria for Chemical Biology (PLACEBO) at the CeMM Research Center for Molecular Medicine of the Austrian Academy of Sciences.

*Sequencing and initial data processing*

Sequencing was performed on the Illumina HiSeq 2000 platform. Base calls provided by the Illumina Realtime Analysis software were converted into BAM files using Illumina2bam (https://github.com/wtsi-npg/illumina2bam) and subsequently demultiplexed using BamIn-dexDecoder from the same package. Initial quality control was performed using the FastQC

software (http://www.bioinformatics.babraham.ac.uk/projects/fastqc/). All bioinformatic analyses were relative to the hg19/GRCh37 assembly of the human genome.

*DNA methylation data processing*

WGBS reads were aligned using Bismark (Krueger and Andrews, 2011), whereas RRBS reads were aligned with BSMAP/RRBSMAP (Xi et al., 2012). Preprocessing of the sequenced reads comprising quality trimming and adapter removal was done with Trimmomatic (Bolger et al., 2014) in both cases, and DNA methylation calling was done with Bismark for WGBS data and with Bis-SNP for RRBS data (Liu et al., 2012). We measured the concordance between biological replicates by dividing the genome into 1-kilobase tiling regions and calculating the correlation across replicates of the average DNA methylation level in each region. We then combined WGBS and RRBS to obtain a set with high coverage in CpG islands as well as uniform baseline coverage genome-wide. With this combined dataset for both the EWS-FLI1 high and the EWS-FLI1 low state, we calculated total CpG coverage for different region types: (i) a set of CpG Islands obtained from the UCSC Genome Browser; (ii) promoter regions defined as the 2 kilobases upstream of TSSs according to our gene model analysis (Figure S3); and (iii) 1-kilobase tiles throughout the genome. To estimate average coverage, for each region set we summed the number of reads covering each CpG and then divided by the number of CpGs in the region. RnBeads (Assenov et al., 2014) was used for quality control and initial analysis of the DNA methylation data (Bock, 2012), while all additional analyses were performed in R (http://r-project.org/)

*Histone modification data processing*

ChIP-seq reads were aligned with Bowtie2 (Langmead et al., 2009). MACS2 (Zhang et al., 2008) was used for peak calling in each biological replicate with a pooled input dataset as baseline. The MACS2 bdgcmp tool was used in 'subtract' mode to plot a signal track for visual inspection via a UCSC Genome Browser track hub. Finally, the R/Bioconductor package diffBind (http://www.bioconductor.org/packages/release/bioc/html/DiffBind.html) was used in combination with custom R scripts to identify peak regions that show significant differences in binding occupancy. To assess the relationships among the histone marks, we extracted average signal scores in 1-kilobase tiling regions across the genome, and hierarchically clustered all the histone data.

*ATAC-seq data processing*

ATAC-seq reads were aligned with Bowtie2 (Langmead et al., 2009). After shifting the reads as described in the original protocol (Buenrostro et al., 2013), we created smoothed density tracks and called peaks using dipPeak (http://dippeak.computational-epigenetics.org), a kernel density estimator.

*Gene expression data processing*

RNA-seq reads were aligned with TopHat2 (Kim et al., 2013). We used the Ensembl transcript set (Homo sapiens, e73, September 2013) as reference transcriptome and processed each biological replicate separately. Cufflinks (Trapnell et al., 2013) was used to assemble transcripts from the spliced read alignments, again with the Ensembl transcriptome as refer-

ence. We allowed *de novo* assembly of transcript models in a first round of the analysis. The resulting custom transcript and gene set served as the basis throughout the rest of the study. When assessing differential expression with Cuffdiff, the RNA-seq of all relevant biological replicates were combining using Cuffmerge. Finally, the R/Bioconductor packages cummerbund (http://www.bioconductor.org/packages/release/bioc/html/cummeRbund.html) and biomaRt (http://www.bioconductor.org/packages/release/bioc/html/biomaRt.html) were used in custom R scripts to perform quality assessment and to refine the analysis results. In a second stage of the analysis, we profiled the transcriptomes after drug treatment. To keep the set of transcripts consistent throughout the analysis, the assembled and merged transcripts of the first stage were used as the reference for the Cufflinks transcriptome assembly step, this time suppressing de novo transcript assembly.

*Composite histone signal*

For Figure 2 and Figure S2, we aggregated histone ChIP-seq signal across regions centered at TSSs and at EWS-FLI1 binding sites. We extracted ChIP-seq signals for each histone mark in 5-kilobase windows surrounding each TSS. We divided TSSs in two ways: first, by expression level (high: top 20%; low: bottom 10%; mid: everything else); and second, by distance to the nearest EWS-FLI1 binding site (proximal: <5kb; distal: 5kb-40kb; none: >40kb). Cutoffs of 5kb and 40kb are reasonable arbitrary values chosen to roughly balance the number of genes in each category. We expect these cutoffs to capture the difference between binding sites that are obviously tied to a particular TSS (<5kb away) versus those that are still reasonably close, but less clear in their TSS assignment and more likely to be associated with distal histone modifications. We then plotted aggregate signal intensity surrounding the TSS for each of these groups of TSSs. To assess histone patterns at EWS-FLI1 binding sites, we aggregated ChIP-seq signal centered on the binding sites instead of the TSSs, dividing the sites by the distance to the nearest TSS.

*Clustering genes based on epigenetic profile*

To consider genes from the perspective of transcription start sites, we started with the TSS models output from cuffmerge (as described above). Because we have additional data about transcription initiation through the histone marks (H3K4me3), which is not considered by cufflinks, we first filtered the cufflinks TSSs by requiring that there be a MACS2-called peak of H3K4me3 in either the EWS-FLI1 high state or the EWS-FLI1 low state within 2.5 kilobases of the TSS. This produced a high-confidence set of 26,750 TSSs that are active in at least one of the two conditions we considered.

We then annotated each TSS for the status of each histone mark by assigning a single score summarizing the strength of each histone mark at each TSS. For histone marks generally found in core promoters (H3K4me3, H3K27ac, H3K56ac), we took the maximum peak score within a 1-kilobase window symmetrically surrounding the TSS. For H3Kme1, which is known to be more dispersed surrounding the TSS (Calo and Wysocka, 2013), we used a larger window of 5 kilobases. We also assessed H3K27me3 in 1kb windows at TSSs for this matrix. This resulted in a matrix of genes-by-modifications, and these five histone marks were used to cluster the TSSs.

We used a two-step approach to define the clusters. In the first step, we used an unsupervised hierarchical clustering on a weighted discrete version of the histone scores. We first

discretized the scores into 3 classes: absent (no peak present; class 0), peak (class 1), or strong peak (at least 2 standard deviations above average; class 2). H3K27me3 was restricted to 2 classes, as the intensity of this signal is assumed to be less relevant than its presence/absence. We assigned the absent class a score of 0, peaks to 1.5, and strong peaks to 2, and then weighted these discrete scores as follows: H3K27me3: 3; H3K4me3: 1.5; H3K4me1: 1; H3K56ac: 1; H3K27ac: 1. We observed that this weighting strategy assigns biological relevance to the histone marks, resulting in biologically meaningful clusters; for example, the heavy weight on H3K27me3 encourages the algorithm to preferentially cluster TSSs with this mark together.

To assess the strength of these cluster assignments, we fed the result of the unsupervised hierarchical clustering into a multinomial logistic regression. We used the glmnet R package, using a leave-one-out cross-validation to select a lambda. This effectively assigned a confidence score to each cluster assignment. We then selected a few of the top-scoring TSSs from each cluster to examine, and then manually classified these genes into “expert clusters”. During the course of manual classification, we settled on four distinct clusters corresponding to four regulation modes. We classified 40-50 genes from each of the two experimental conditions, resulting in about 8-12 example genes for each expert-assigned cluster (Supplemental Website).

We used these expert-assigned cases to build models of the four regulation clusters we had observed. We used the same multinomial logistic regression strategy described above, fitting a lasso model to the expert examples. The training set performs almost perfectly in the cross-validation steps used to fit the model, implying high confidence in the cohesion of the clusters (Figure S3). We then used this model to predict the cluster assignment of the remaining genes that were not evaluated by the expert cluster assignment. We set a differential expression q-value cutoff of 0.2, and predicted cluster assignment for all genes that met this threshold. The confidence of the resulting predictions is general, and to further refine the list, we restrict the final set to only genes that are predicted to belong to a single class with at least 95% probability. This procedure was repeated independently for EWS-FLI1 correlated and EWS-FLI1 anticorrelated genes; with the correlated genes being clustered on the basis of their histone patterns in the EWS-FLI1 low state, and the anticorrelated genes on the basis of their histone patterns in the EWS-FLI1 high state.

*Annotating clusters for enrichment*

To identify any functional similarities within each cluster of genes we identified, we developed a comprehensive enrichment analysis tool that detects significant overlaps between a given set of genes (and their promoter regions) and several manually curated databases, including MSigDB (Liberzon et al., 2011), Cistrome (Liu et al., 2011), DNase hypersensitivity (Sheffield and Furey, 2012; Sheffield et al., 2013), and ENCODE transcription factor binding datasets (ENCODE Project Consortium, 2012). For this analysis we employed two different types of comparison: First, using a more traditional enrichment analysis, we compared sets of gene names to gene name sets in MSigDB. We also developed a location-based enrichment tool, Location Overlap Analysis (LOLA; http://lola.computational-epigenetics.org), to allow us to compare loci of interest to published sets of genomic regions, such as ChIP-seq results. In this setting, we avoided assigning TSSs to specific gene names, instead considering just the location of the TSS and looking for enrichment of other datasets within these regions. For the location-based enrichments, we tested for significant overlap with several hand-curated region set databases. We downloaded data from Cistrome, ENCODE transcription factor ex-

periments, and ENCODE DNase hypersensitivity tissue-specificity assignments. We used Fisher's exact tests to obtain p-values for each comparison, and ranked comparisons by p-value to identify the gene sets with most significant overlap.

Using this enrichment tool, we searched for significant enrichments in three different comparisons. First, we compared our cluster-assigned genes to the entire set of genes identified by the RNA-seq analysis. This resulted in general Ewing sarcoma genes, confirming that our differential genes match previous definitions of changes induced by EWS-FLI1 (Supplemental Website). Second, we searched for enrichments in each cluster versus genes in the other clusters; in other words, we restricted the universe of possible genes to only those regulated by EWS-FLI1. This identified cluster-specific effects (Figure 4; Supplemental Website). Finally, we tested for enrichments for the EWS-FLI1 correlated genes against EWS-FLI1 anticorrelated genes by restricting the universe of possible genes to those regulated in either direction within the same cluster (Figure 4; Supplemental Website).

*Enhancers, super-enhancers, and tissue-specificity*

To assess the genomic context of differential H3K27ac peaks, we used the set of differential H3K27ac peaks created by diffBind. We defined promoters as 2 kilobases upstream of TSSs, with TSSs defined by our merged annotation based on the RNA-seq data we generated. We then annotated the peaks as promoters if they overlapped an H3K4me3 peak; otherwise, we classified them as distal enhancer elements, because H3K27ac is commonly used as the defining mark for active enhancers.

We used ROSE to search for super-enhancers based on our H3K27ac data (Loven et al., 2013; Whyte et al., 2013). We assembled a set of super-enhancers for each of the two replicates of EWS-FLI1 expressing cells (EWS-FLI1 high state). To assemble a final list of super-enhancers we combined the replicates by requiring that a super-enhancer is present (overlapping) in both replicates. We used these definitions of super-enhancers to test for overlap with differential H3K27ac peaks. After defining super-enhancers, we subdivided the set of enhancers into those that overlap a super-enhancer, and those that overlap an EWS-FLI1 binding peak. After defining super-enhancers, we annotated them for nearby genes. We listed all genes within 50 kilobases of the super-enhancer, and selected a few promising candidates to highlight in the main figure. To assess tissue specificity, we used DNase hypersensitivity data from 72 human cell types published previously (Sheffield et al., 2013). For each differential H3K27ac peak, we calculated the number of samples that have a normalized DNase hypersensitivity score above a cutoff (0.4) within the H3K27ac peak. We then averaged this value across all peaks of a certain type, and looked at the relationship between average values. To calculate p-values, we used Kolmogorov-Smirnov tests on the distribution of average tissue-specificities.

*DNA methylation and EWS-FLI1 binding*

We divided H3K27ac peaks into those bound or not bound by EWS-FLI1. We then compared the distribution of average DNA methylation level between these sets of regions. We repeated this test for both EWS-FLI1 high and EWS-FLI1 low conditions, and found similar results. To confirm, we next compared DNA methylation signal before and after EWS-FLI1 knockdown. We considered all H3K27ac peaks with at least 25 total measurements of CpG methylation in each condition. We used a Fisher's exact test to identify significant difference be-

tween the conditions, and then looked at the absolute difference in methylation between the conditions for those with significant difference. For the corresponding comparison in H3K27ac data, we looked at the distribution of absolute fold-change for regions with significant difference in H3K27ac (with significance defined by diffBind, as explained previously). This analysis showed that while H3K27ac change is highly (and directionally) affected by EWS-FLI1 binding, DNA methylation change is not.

## Supplemental References


Assenov, Y., Müller, F., Lutsik, P., Walter, J., Lengauer, T., and Bock, C. (2014). Comprehensive analysis of DNA methylation data with RnBeads. Nat Methods *11*, 1138-1140.

Ban, J., Bennani-Baiti, I.M., Kauer, M., Schaefer, K.L., Poremba, C., Jug, G., Schwentner, R., Smrzka, O., Muehlbacher, K., Aryee, D.N., *et al.* (2008). EWS-FLI1 suppresses NOTCH-activated p53 in Ewing's sarcoma. Cancer Res *68*, 7100-7109.

Bock, C. (2012). Analysing and interpreting DNA methylation data. Nat Reviews Genet *13*, 705-719.

Bolger, A.M., Lohse, M., and Usadel, B. (2014). Trimmomatic: a flexible trimmer for Illumina sequence data. Bioinformatics *30*, 2114-2120.

Buenrostro, J.D., Giresi, P.G., Zaba, L.C., Chang, H.Y., and Greenleaf, W.J. (2013). Transposition of native chromatin for fast and sensitive epigenomic profiling of open chromatin, DNA-binding proteins and nucleosome position. Nat Methods *10*, 1213-1218.

Calo, E., and Wysocka, J. (2013). Modification of enhancer chromatin: what, how, and why? Mol Cell *49*, 825-837.

Carrillo, J., Garcia-Aragoncillo, E., Azorin, D., Agra, N., Sastre, A., Gonzalez-Mediero, I., Garcia-Miguel, P., Pestana, A., Gallego, S., Segura, D., *et al.* (2007). Cholecystokinin down-regulation by RNA interference impairs Ewing tumor growth. Clin Cancer Res *13*, 2429-2440.

ENCODE Project Consortium (2012). An integrated encyclopedia of DNA elements in the human genome. Nature *489*, 57-74.

Kim, D., Pertea, G., Trapnell, C., Pimentel, H., Kelley, R., and Salzberg, S.L. (2013). TopHat2: accurate alignment of transcriptomes in the presence of insertions, deletions and gene fusions. Genome Biol *14*, R36.

Krueger, F., and Andrews, S.R. (2011). Bismark: a flexible aligner and methylation caller for bisulfite-seq applications. Bioinformatics *27*, 1571-1572.

Langmead, B., Trapnell, C., Pop, M., and Salzberg, S.L. (2009). Ultrafast and memory-efficient alignment of short DNA sequences to the human genome. Genome Biol *10*, R25.

Liberzon, A., Subramanian, A., Pinchback, R., Thorvaldsdottir, H., Tamayo, P., and Mesirov, J.P. (2011). Molecular signatures database (MSigDB) 3.0. Bioinformatics *27*, 1739-1740.

Liu, T., Ortiz, J.A., Taing, L., Meyer, C.A., Lee, B., Zhang, Y., Shin, H., Wong, S.S., Ma, J., Lei, Y., *et al.* (2011). Cistrome: an integrative platform for transcriptional regulation studies. Genome Biol *12*, R83.

Liu, Y., Siegmund, K.D., Laird, P.W., and Berman, B.P. (2012). Bis-SNP: combined DNA methylation and SNP calling for Bisulfite-seq data. Genome Biol *13*, R61.

Loven, J., Hoke, H.A., Lin, C.Y., Lau, A., Orlando, D.A., Vakoc, C.R., Bradner, J.E., Lee, T.I., and Young, R.A. (2013). Selective inhibition of tumor oncogenes by disruption of super-enhancers. Cell *153*, 320-334.

Sheffield, N.C., and Furey, T.S. (2012). Identifying and characterizing regulatory sequences in the human genome with chromatin accessibility assays. Genes *3*, 651-670.

Sheffield, N.C., Thurman, R.E., Song, L., Safi, A., Stamatoyannopoulos, J.A., Lenhard, B., Crawford, G.E., and Furey, T.S. (2013). Patterns of regulatory activity across diverse human cell types predict tissue identity, transcription factor binding, and long-range interactions. Genome Res *23*, 777-788.

Trapnell, C., Hendrickson, D.G., Sauvageau, M., Goff, L., Rinn, J.L., and Pachter, L. (2013). Differential analysis of gene regulation at transcript resolution with RNA-seq. Nat Biotechnol *31*, 46-53.

Whyte, W.A., Orlando, D.A., Hnisz, D., Abraham, B.J., Lin, C.Y., Kagey, M.H., Rahl, P.B., Lee, T.I., and Young, R.A. (2013). Master transcription factors and mediator establish super-enhancers at key cell identity genes. Cell *153*, 307-319.

Xi, Y., Bock, C., Müller, F., Sun, D., Meissner, A., and Li, W. (2012). RRBSMAP: a fast, accurate and user-friendly alignment tool for reduced representation bisulfite sequencing. Bioinformatics *28*, 430-432.

Zhang, Y., Liu, T., Meyer, C.A., Eeckhoute, J., Johnson, D.S., Bernstein, B.E., Nusbaum, C., Myers, R.M., Brown, M., Li, W., *et al.* (2008). Model-based analysis of ChIP-Seq (MACS). Genome Biol *9*, R137.
